# Neurosteroid Actions in Memory and Neurologic/Neuropsychiatric Disorders

**DOI:** 10.3389/fendo.2019.00169

**Published:** 2019-04-09

**Authors:** Marcia H. Ratner, Vidhya Kumaresan, David H. Farb

**Affiliations:** Laboratory of Molecular Neurobiology, Department of Pharmacology and Experimental Therapeutics, Boston University School of Medicine, Boston, MA, United States

**Keywords:** neurosteroid, memory, anxiety, depression, schizophrenia, allopregnanolone, pregnenolone sulfate, Alzheimer's disease

## Abstract

Memory dysfunction is a symptomatic feature of many neurologic and neuropsychiatric disorders; however, the basic underlying mechanisms of memory and altered states of circuitry function associated with disorders of memory remain a vast unexplored territory. The initial discovery of endogenous neurosteroids triggered a quest to elucidate their role as neuromodulators in normal and diseased brain function. In this review, based on the perspective of our own research, the advances leading to the discovery of positive and negative neurosteroid allosteric modulators of GABA type-A (GABA_A_), NMDA, and non-NMDA type glutamate receptors are brought together in a historical and conceptual framework. We extend the analysis toward a state-of-the art view of how neurosteroid modulation of neural circuitry function may affect memory and memory deficits. By aggregating the results from multiple laboratories using both animal models for disease and human clinical research on neuropsychiatric and age-related neurodegenerative disorders, elements of a circuitry level view begins to emerge. Lastly, the effects of both endogenously active and exogenously administered neurosteroids on neural networks across the life span of women and men point to a possible underlying pharmacological connectome by which these neuromodulators might act to modulate memory across diverse altered states of mind.

## Introduction

A major question in neuroscience since the initial discovery that somatically released gut peptides could alter central nervous system (CNS) function relates to whether and how the body can influence or modulate brain function. The science of neuroendocrinology was advanced conceptually 50 years ago by the independent discoveries of Schally, Leeman and Reichlin, demonstrating that the gut peptides thyrotropin-releasing hormone (TRH) (1, 2) and substance P (3, 4) were synthesized, stored, and released in the hypothalamus as endogenous neuromodulators. The demonstration of local synthesis of neuropeptides within the CNS presented a non-canonical mechanism for gut peptides to act as chemical neurotransmitters at synapses, without transport via the systemic circulation and transport across the blood-brain barrier (BBB).

The BBB does not impair access of sex steroids to the CNS to the same extent as gut peptides. Lipophilic steroid hormones, such as progesterone, estradiol and testosterone cross the BBB and readily gain access to the CNS (5) where they can serve as agonists of steroid hormone receptors that in turn act at genomic response elements. In the early 1980s, several lines of evidence from Etienne, Baulieu, and Robel (6–9) challenged the central dogma that neuroactive steroids were exclusively synthesized peripherally, demonstrating for the first time that steroids could be synthesized from cholesterol within the CNS.

Such steroids were called *neurosteroids* and an intensive search began to identify which steroids belonged to this group and to define their function. An early clue came from the research of Selye (10) showing that steroids could have anesthetic effects. Four decades later, in 1983, radiolabeling studies by Sapolsky, McEwen, and Rainbow revealed uptake of corticosterone in the stratum oriens and apical dendrite regions of the hippocampus, suggesting that GABAergic interneurons in these regions might possess corticosterone receptors (11). Corticosterone treatment had been shown to affect GABA uptake in the hippocampus, possibly suggesting a mechanism for hormonal modulation of memory. In a seemingly unrelated study, while investigating the pharmacological mechanism of action of the synthetic steroid anesthetic alphaxalone, Harrison and Simmonds (12) demonstrated that alphaxalone and barbiturates shared a common mechanism of action via augmenting GABA_A_R action. Subsequent research by multiple investigators demonstrated that several reduced metabolites of progesterone and deoxycorticosterone act as positive allosteric modulators of GABA_A_Rs (13–17), much like benzodiazepines (18, 19). Other research (20, 21) also suggested that neurosteroids might be capable of modulating inhibitory GABAergic neurotransmission.

**Figure 1:**
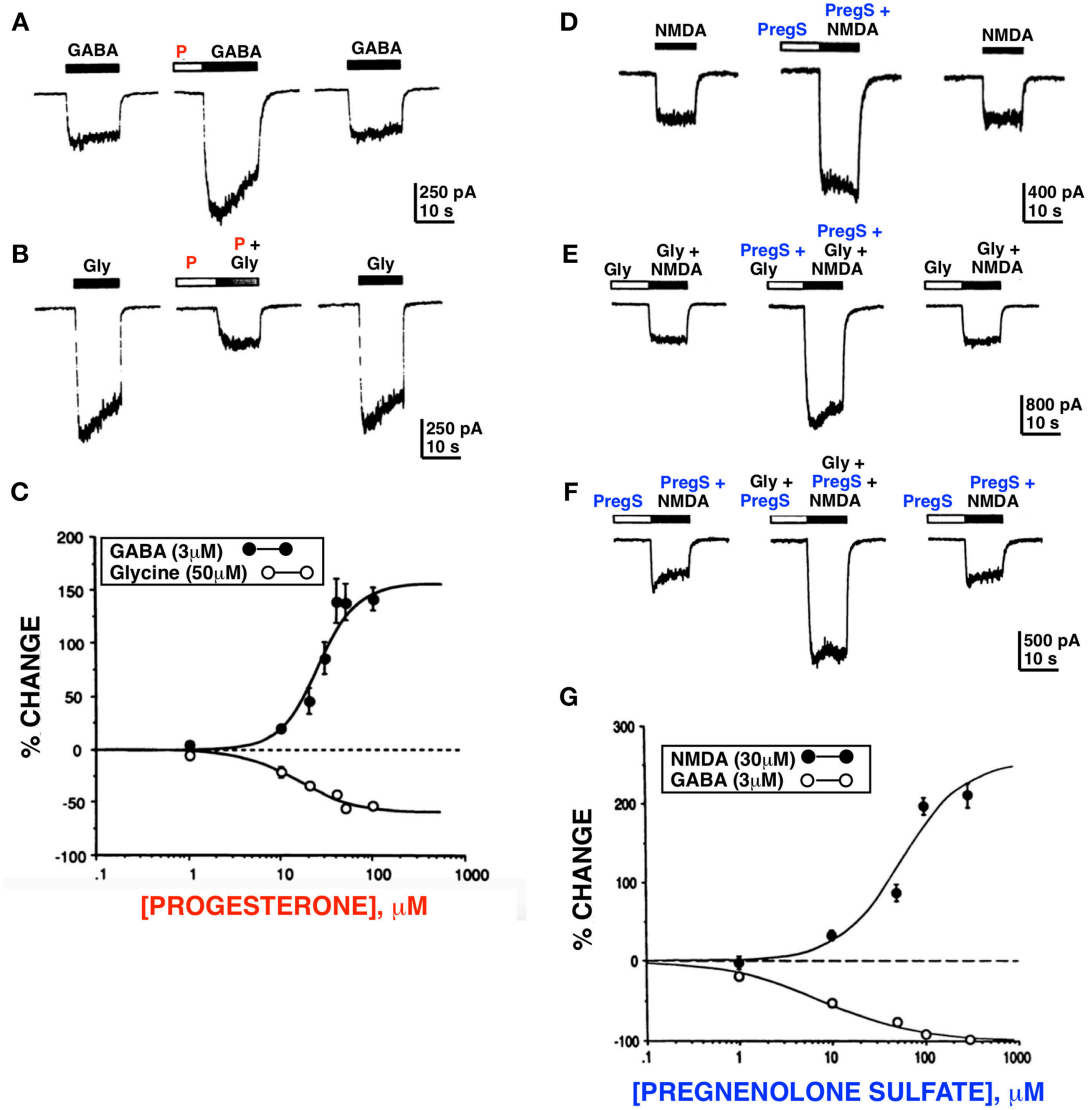
Progesterone and PregS differentially modulate whole cell currents induced by GABA, glycine and NMDA. Progesterone (P) (100 μM) potentiates the GABA response **(A)** and inhibits the glycine **(B)** response. **(C)** Dose response curves for progesterone modulation of GABA and glycine currents; enhancement of the GABA response by progesterone occurs over the same concentration range as inhibition of the glycine response. **(D)** PregS (100 μM) potentiates the NMDA response (normal media [Gly]). **(E)** PregS and glycine potentiate NMDA response by different mechanisms. **(F)** In the presence of the maximal concentration (10 μM) of glycine, PregS (100 μM) enhances (179 ± 17.1%; *n* = 4) the response induced by 30 μM of NMDA; **(F)** In the presence of near maximal concentration of PregS (100 μM), glycine (10 μM) reversibly potentiates (210 ± 36.5%; *n* = 4) the NMDA response. **(G)** Dose response curves for PregS modulation of NMDA and GABA currents. Enhancement of the NMDA response by PregS occurs over the same concentration range as inhibition of the GABA response (*Horizontal bar above each trace represents period of drug application*) [Modified from Wu et al. (23, 24) with Permission].

As new ideas emerged from clinical studies by Andrew Herzog in the mid 1980s concerning the possible role of estrogen and progesterone in catamenial epilepsy (22), we hypothesized that progesterone might act as a positive allosteric modulator of the GABA_A_R. This led to the early work of Fong-sen Wu and Terrell Gibbs in my lab (23) showing that progesterone did in fact modulate GABA_A_ and glycine receptors. Unexpectedly, we also found that pregnenolone sulfate (PregS), a novel negatively charged steroid derived from the sulfation of pregnenolone (PREG), potentiated N-methyl-D-aspartate receptor (NMDAR) function (24) (Figure 1 and Table 1).

**Table 1:** Historical discoveries in pregnene series neurosteroids.

**References**	**System and methodology**	**Key findings and novel outcomes**
Majewska and Schwartz (21); Majewska, (25)	Measurement of inhibition of GABA-mediated uptake of labeled chloride in rat brain synaptosome preparations	Demonstration of a possible receptor target for PregS as an inhibitor of GABA_A_ receptors
Wu et al. (24); Farb et al. (26)	Whole cell patch clamp of NMDARs currents in cultured chick spinal cord neurons	PregS and related sulfated neurosteroids of the pregnene series potentiate NMDARs acting as functional neuromodulators in glutamatergic synaptic transmission
Irwin et al. (27)	Micro-spectrofluorimetric measurement of intracellular calcium in primary neuronal cultures of rat hippocampus	Neurosteroids, such as PregS modulate excitation-inhibition balance in the CNS
Flood et al. (28–30); Plescia et al. (31); Plescia et al. (32); Abdel-Hafiz, (33)	Behavioral assays in rodent models	Pregnene group neurosteroid-mediated enhancement of cognitive function. Subsequent studies provide further demonstration that PREG and its metabolite, PregS, enhances memory.
Park-Chung et al. (34)	Whole cell patch clamp in cultured chick spinal cord neurons	Elucidation of subunit-specific effects of PregS and demonstration that pregnene neurosteroids modulate excitatory ionotropic GluRs.
Park-Chung et al. (35, 36)	Whole cell recordings in cultured chick spinal cord neurons. Structure-activity studies using recordings from recombinant NMDAR expressed in *Xenopus* oocytes	Identification of PregS binding site. First demonstration that steroids function by binding to an extracellular site on NMDAR.
Yaghoubi et al. (37); Malayev et al. (38); Cameron et al. (39)	Voltage clamp recordings of recombinant NMDAR in *Xenopus* oocytes. Bacterial cultures. Intrinsic fluorescence spectroscopy.	PregS positively modulates GluN2A- and GluN2B-containing NMDARs. PregS inhibits GluN2C- and GluN2D-containing NMDARs and AMPA/kainate receptors.
Partridge and Valenzuela, (40); Sliwinski et al. (41); Sabeti et al. (42)	Measurement of long-term potentiation using hippocampal slice electrophysiology	PregS modulates synaptic strength critical for learning and memory. nM PregS: modulates LTP via NMDARs; modulates presynaptic release of glutamate; voltage-gated Ca^2+^ channel induced LTP potentiation.
Jang et al. (43); Horak et al. (44); Kostakis et al. (45)	Electrophysiology; molecular modeling; recombinant chimeric NMDARs, with altered residues by means of site directed mutagenesis expressed in *Xenopus* oocytes.	PregS exhibits a rich modulatory repertoire enabled by the structural diversity of NMDARs. The extracellular steroid-modulatory site (SMD1) contains the J/K helices and contiguous TMD4. Extracellular loop between TMD3 and 4 mediates both excitatory and inhibitory effects.
Petrovic et al. (46)	Voltage-clamp studies in HEK293 cells expressing NR1/NR2B NMDARs and cultured rat hippocampal neurons.	PregS influences NMDAR-dependent responses via a phosphorylation dependent mechanism.
Kostakis et al. (47); Smith et al. (48)	Whole cell recordings of recombinant receptors expressed in oocytes and [Ca^2+^]i imaging using and primary neuronal cultures of embryonic cortical neurons	First demonstration that physiologically relevant concentrations of PregS modulate synaptic plasticity *in vitro*. Picomolar concentrations are sufficient to increase intracellular Ca^2+.^ Increased intracellular Ca^2+^ increases surface GluN1-NMDARs and CREB activation. PregS mediated modulation of NMDARs results in delayed onset potentiation occurs via a non-canonical G-protein and Ca^2+^ dependent manner. This potentiation is absent when the J/K helices and TMD4 of GluN2B are replaced with that of GluN2D further establishing the subunit-dependent action of PregS and importantly the extracellular binding site of PregS.
Smith et al. (48); Adamusová et al. (49)	[Ca^2+^]i imaging studies using primary rat hippocampal neuronal cultures and HEK293 cells	Picomolar to femtomolar concentrations of PregS increases intracellular Ca2^+^
Marx et al. (50–52); Ritsner et al. (53)	Human subjects for clinical effects	Adjunctive treatment with PREG in schizophrenia and schizoaffective disorders shown to reduce negative symptoms and improve positive symptoms of verbal memory and attention. Post-treatment elevation of ALLO and PregS correlate with enhancement of cognitive function. Metabolism of PREG to PregS likely ameliorating NMDAR hypofunction implicated in schizophrenia.
Wilding et al. (54)	Whole cell recordings and molecular modeling using recombinant chimeric GluN and GluK2 receptor constructs in HEK 293 cells	Confirmation of binding sites and relationship to specific receptor domains elucidated. Confirmed extracellularly directed binding site for PregS. Requirement of TMD likely for pore formation.
Paul et al. (55); Linsenbardt et al. (56)	Investigations of synthetic PregS analogs and oxysterols as therapeutics using *in vitro* and *ex vivo* electrophysiological methods	The major brain-derived cholesterol metabolite, 24(S)-hydroxy cholesterol modulates NMDARs by binding to an intracellular site. This intracellular oxysterol binding site is distinct from the extracellular site that bind PregS.
Vyklicky et al. (57)	Electrophysiological investigations of *de novo* missense mutations of the hGluN2B expressed in HEK cells.	Missense mutations of the hGluN2B subunit located in membrane domains lead to multiple defects that manifest by the NMDAR loss of function that can be rectified by steroids.
Chisari et al. (58)	Hippocampal slice electrophysiology, *in vitro* electrophysiological recordings from cultured hippocampal neurons and *Xenopus* oocytes. expressing recombinant NMDARs.	Analogs of PregS and oxysterols, such as KK169 shown to exhibit properties of PregS.

Over the ensuing 25 years, endogenous neurosteroids have been implicated in learning and memory function, hippocampal information processes, and synaptic plasticity (28, 29, 48, 59–63). Neurosteroids have also been implicated in the etiology and treatment of learning and memory disturbances associated with certain neuropsychiatric disorders, including schizophrenia, depression, and anxiety (50, 64–66) (Table 2).

**Table 2:** Neurosteroids in human neurologic and neuropsychiatric disorders.

**Disorder**	**Neurosteroid(s)**	**Clinical response**	**Memory**
**ALZHEIMER'S DISEASE**			
Temporal cortex: Naylor et al. (67)	Increased DHEA and PREG decrease ALLO levels	ALLO levels inversely correlate with Braak and Braak neuropathological stage	NR
Striatum and cerebellum: Hypothalamus: Weill-Engerer et al. (60)	Low PregS and DHEAS Low DHEAS	Negative correlation between cortical β-amyloid and PregS in striatum and cerebellum Negative correlation between levels of pTau and DHEAS	NR
DHEAS: Carlson et al. (68)	Increases in plasma	AD risk not linked with DHEAS	Increased memory performance
Cortisol: Csernansky et al. (69)	Increases in plasma	More rapid disease progression	Increased memory performance
Cortisol: Carlson et al. (68)	Decreases in plasma	No relationship to AD risk	Increased Delayed Route Recall
DHEA in women: Rasmuson et al. (70)	Increases in serum	Associated with AD risk	NR
DHEA and DHEAS in men: Aldred and Mecocci (71)	Decreases in plasma	Associated with AD risk	NR
Cortisol in men: Rasmuson et al. (70)	Increases in serum	Associated with AD risk	NR
aMCI in men: Cherrier et al. (72)	Testosterone treatment	Reduced depression	Improved verbal memory
**MOOD DISORDERS**			
GAD in elderly: Mantella et al. (73)	Increased saliva cortisol	Positive correlation between symptoms and saliva cortisol	NR
Generalized social phobia in men: Heydari and Le Mellédo (74)	Decreased plasma PregS	PregS levels lower in generalized social phobia subjects	NR
PTSD in women: Rasmusson et al. (75)	Decreased CSF ALLO	ALLO/DHEA correlates negatively with PTSD and Profile of Mood States depression dejection scores	NR
PTSD in men: Rasmusson et al. (76)	ALLO and pregnanolone CSF	Negative correlation between ALLO + pregnanolone and symptoms severity	NR
Acute stress: Droogleever Fortuyn et al. (77)	Increased plasma ALLO	Peripheral benzodiazepine receptor density increased in blood platelets	NR
Acute psychosocial stress in elderly: Wolf et al. (78)	DHEA at 50 mg/kg/day for 2 weeks	DHEAS lower than in young adults. DHEA replacement increases DHEAS	Enhanced attention; Impaired declarative memory and recall, but not spatial memory.
Dysphoria: Premenstrual Girdler et al. (79)	Increased plasma ALLO/progesterone	Greater levels of premenstrual anxiety	NR
Post-partum depression: Kanes et al. (80, 81)	ALLO	Reduction in hamilton depression rating scale scores.	NR
**SCHIZOPHRENIA**			
Marx et al. (50)	Adjunctive PREG	Improves negative symptoms and ameliorates cognitive deficits	NR
Marx et al. (51)	Treatment with PREG for 8 weeks	Increases serum PREG and its metabolites ALLO and PregS	Increased serum PREG aligns with BACS score
**CATAMENIAL EPILEPSY**			
Herzog (22) Herzog and Frye (82)	Progesterone ALLO	Associated with progesterone No association between serum ALLO and seizure frequencies in women treated with progesterone stratified by catamenial vs. non-catamenial epilepsy Serum ALLO correlated with seizure reduction in progesterone-treated women who reported a 3-fold or greater perimenstrual increase in average daily seizure frequency	NR
Partial intractable epilepsy: Valencia-Sanchez et al. (83)	Adjunctive progesterone	No effect on catamenial or non-catamenial seizures	NR

*NR, not reported*.

Memory dysfunction is frequently comorbid with age-related neurodegenerative diseases, such as Alzheimer's disease (AD) (84). From a therapeutic standpoint, the lack of an effective treatment for memory disorders extends beyond neurodegeneration to a wide range of neuropsychiatric disorders, such as depression and schizoprhenia.

Memory dysfunction seriously impacts performance of routine tasks necessary for a productive and healthy life, including the ability to maintain gainful employment and compliance with treatment plans (85). This review summarizes the field from the perspective of our own research, which has spanned the past three decades, and attempts to bring together state-of-the-art findings related to the role of neurosteroids in memory dysfunction, as seen in patients with schizophrenia, depression, and anxiety disorders. We believe that a greater understanding of how steroids modulate neural network activity will help lay the foundation for a unifying theory of neurosteroid action in the brain centered on a systems level “pharmacological connectome.”

## Synthesis, Structure, Transport and Cellular Targets of Neurosteroids

### Synthesis and Translocation

Neurosteroid synthesis involves translocation of cholesterol across the mitochondrial membrane by transport proteins, such as the steroidogenic acute regulatory protein (StAR protein), the translocator protein (TSPO), voltage-dependent anion channel (VDAC) protein and the adenine nucleotide transporter (ANT) protein (86–89). The conversion of cholesterol to PREG is catalyzed by the enzyme cytochrome P450 side chain cleavage (P450 scc) located on chromosome 15 in humans (90). Other enzymes that play a role in the biosynthesis of neurosteroids include 5α-reductase and 3α-hydroxysteroid dehydrogenase (91–93). These two enzymes are involved in the biosynthesis of allopregnanolone (ALLO) and tetrahydrodeoxycorticosterone (THDOC); the identification of neurons that express these enzymes in the rodent cerebral cortex, hippocampus, olfactory bulb, amygdala, and thalamus suggests that ALLO and THDOC can be synthesized locally from precursors within the CNS (94).

The sulfation and desulfation of neurosteroids further alters both the pharmacokinetic and pharmacodynamic properties of these endogenous neuromodulators (95). In humans, sulfation of PREG to PregS is catalyzed by SULT2B1a, whereas SULT2B1b preferentially catalyzes the sulfation of 3beta-hydroxysteroids. Non-human primate studies suggest that age-dependent changes in the expression of these enzymes could play a role in age-related changes in cognitive function (96, 97).

Neurosteroids and their sulfated conjugates can be characterized based on their core backbone structures as pregnanes, pregnenes, androstanes, progesterones, and deoxycorticosterones. Neurosteroids in these respective subcategories include: pregnanolone and pregnanolone sulfate; PREG and PregS; dehydroepiandrosterone (DHEA) and dehydroepiandrosterone sulfate (DHEAS); progesterone and ALLO; and, deoxycorticosterone and THDOC (98). While delineating the neurological function of sulfated neurosteroids remains a frontier in neuroendocrinology, some fundamental progress has been made these past few decades.

### Physiological Actions

The physiologic effects of neurosteroids are mediated through direct interactions with neurotransmitter receptors and transporters, and indirectly via promotion of second-messenger signaling cascades (47, 48, 99–103). Their rapid non-genomic effects are exerted via the allosteric modulation of inhibitory and excitatory receptors located in the surface membrane. In some cases, neurosteroids exert genomic effects, at least in part, by activation of intracellular steroid receptors (104). The degree to which neurosteroids produce genomic and non-genomic effects depends on the extent to which they are metabolized (e.g., PREG to progesterone), and the extent to which the parent molecule and its neuroactive metabolites modulate extra- and intracellular receptors (104).

The modulation of GABAergic neurotransmission by neurosteroids is mediated by interactions with allosteric sites on GABA_A_Rs (105–109), and neurosteroids appear to play a role in regulating the expression of specific GABA_A_R subunits (63). Classical uncharged neurosteroids modulate inhibitory GABA receptors and neurotransmission. Neurosteroids that are known to be relatively potent positive modulators of GABAergic neurotransmission include ALLO, pregnanolone, and TDHOC.

PregS is a relatively potent positive allosteric modulator of NMDAR-mediated synaptic transmission, while pregnanolone sulfate is a relatively potent negative allosteric modulator of NMDAR-mediated glutamatergic neurotransmission (24, 35, 110).

PregS is the most widely studied neurosteroid that potentiates NMDARs (111, 112). 17-hydroxy-PREG is metabolized to DHEA by cytochrome P450 17α-hydroxylase/17,20-lyase. The sulfated form of DHEA, like the sulfated form of PREG (i.e., PregS), is also an NMDAR potentiator. Electrophysiology studies of recombinant NMDARs expressed in *Xenopus* oocytes have established that the effects of PregS are dependent on NMDAR subunit composition. PregS potentiates GluN2A- and GluN2B-NMDARs, whereas it negatively modulates GluN2C- and GluN2D-NMDARs (38). Long known to be critical for learning and memory, transient activation of NMDARs is required for induction of long-term potentiation (LTP) or strengthening of synaptic transmission, as well as long-term depression (LTD) or weakening of synaptic transmission (113). Activation of NMDARs is crucial for many forms of activity-dependent plasticity responsible for learning and memory in the hippocampus and other brain nuclei (114–118).

**Figure 2:**
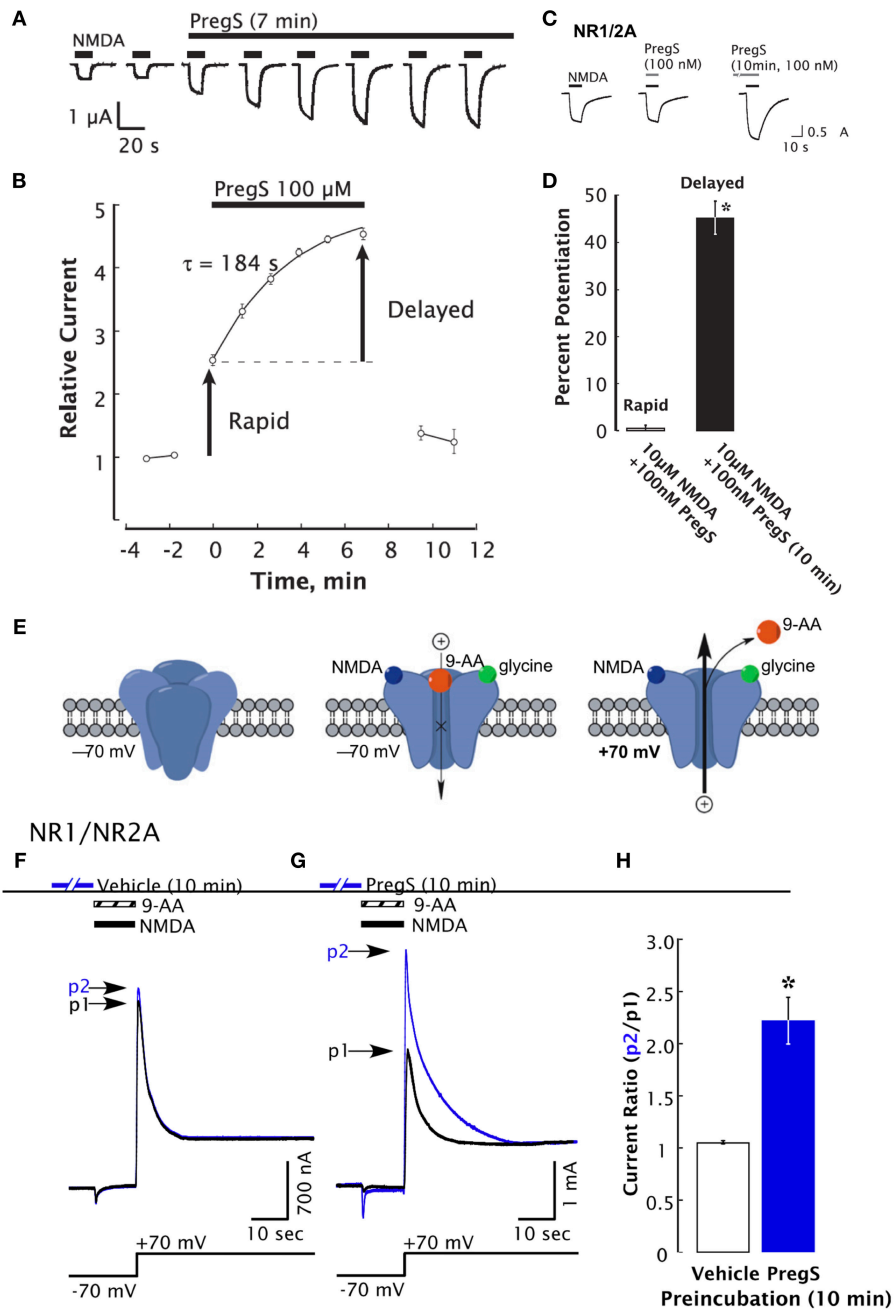
Delayed-onset potentiation of NMDARs is induced by PregS. Two-electrode whole-cell voltage clamp recording from *Xenopus oocytes* expressing NR1/2A subunits following application of PregS (100 μM) and NMDA (300 μM). **(A)** Potentiation of the NMDA response by PregS. Black bars indicate successive applications of NMDA. **(B)** Peak NMDA-induced currents determined as in **(A)** are normalized to the average response before application of PregS beginning at *t* = 0. Smooth curve reflects an exponential fit (*t* = 184 s). Error bars indicate S.E.M. (*n* = 3). Arrows indicate rapid and delayed components of potentiation. **(D)** In oocytes expressing NR1/2A receptors, delayed potentiation but not rapid potentiation is induced by 100 nM PregS. **(D)** Averaged values of normalized peak current responses for rapid and delayed increase. When added simultaneously with NMDA, PregS produces a negligible potentiation of the NMDA response (1 ± 2%), whereas after 10 min pre-incubation with 100 nM PregS, the response to NMDA was enhanced by 45 ± 3%. Error bars represent S.E.M (*n* = 8–10). ^*^Indicates a significant difference between rapid and delayed potentiation (*P* < 0.0005). **(E)** Cartoon shows NMDA and 9-aminoacridine (9-AA) (100 μM) co-applied to *Xenopus oocytes* expressing NR1/2A receptors results in a transient inward current as NMDA-activated channels are blocked by 9-AA (a voltage-dependent open-channel blocker). As the holding potential is switched from −70 to +70 mV, an outward tail current reflecting 9-AA unblock of NMDAR channels ensues (black traces). Cells were then exposed to vehicle (Ba-Ringer) **(F)** or PregS **(G)** for 10 min, and the 9-AA block and unblock sequences were repeated (blue traces). Peak tail currents after baseline subtraction are expressed relative to the control current (black trace) from the same cell (p2/p1). **(H)** The peak current ratio p2/p1 for PregS-treated oocytes (blue bar; *n* = 8) is significantly higher than for vehicle-treated oocytes (white bar; *n* = 6). ^*^Relative current *P* < 0.00001, unpaired 2-tail *t*-test [From Kostakis et al. (47) with Permission].

PregS also acts as a negative allosteric modulator of GABA, glycine, kainate, and α-amino-3-hydroxy-5-methyl-4-isoxazolepropionic acid (AMPA) receptors (34). The synthetic analog of PregS, PREG hemisuccinate, and other related PREG derivatives bearing a negative charge, potentiate the NMDA response (119). This observation suggests that positive modulation of NMDARs is not mediated by the sulfate group *per se*. Additional studies using other synthetic analogs of PregS revealed that a negatively charged moiety at the C3 position is, however, essential for positive modulation of NMDARs (35). PregS may also influence NMDAR-dependent responses via a phosphorylation-dependent mechanism (46). Low nanomolar concentrations of PregS induce a delayed onset increase of the neuronal response to NMDA and trafficking of NMDAR to the cell surface through an intracellular Ca^2+^ ([Ca^2+^]i)-dependent and non-canonical mechanism involving G-proteins (47) (Figure 2). Moreover, low picomolar PregS concentrations appear to be sufficient to increase [Ca^2+^]i and CREB phosphorylation (48).

Effects of PregS on NMDARs are diverse (15). NMDARs possess at least two distinct modulatory sites (38). PregS increases the frequency and duration of NMDA-mediated channel opening while it inhibits AMPA and GABA_A_Rs (24, 110). PregS effects are dependent on the subunit composition of NMDARs (45, 120). PregS potentiates recombinant NMDARs with GluN1-1a/GluN2B through a steroid modulating domain in GluN2B that also modulates tonic proton inhibition and is pH independent. PregS-mediated potentiation of GluN-2C-NMDARs is similarly pH-dependent. On the other hand, PregS-mediated potentiation of GluN2A and 2D-NMDARs is enhanced at reduced pH. The presence of GluN1-1b subunit with an N-terminal exon-5 splicing insert modulates the extent of proton-dependent PregS potentiation (43, 45). The differential pH sensitivity of the NMDAR isoforms to modulation by PregS is likely to be critical in view of the importance of proton sensors in CNS health and disease (45). PregS acts at a site distinct from the PregS site and inhibits NMDARs irrespective of subunit composition (35, 38, 46).

PregS increases spontaneous excitatory post-synaptic currents (sEPSC) frequency but not amplitude. This demonstrates PregS-mediated presynaptic regulation of spontaneous glutamate release and points to a potential significant impact of PregS on hippocampal function. Presynaptic transient receptor potential channel (TRP channel) receptor activation by PregS modulates glutamate release and increases sEPSC in acutely isolated hilar neurons of the dentate gyrus, an increase that is blocked by TRP channel antagonists (121). Dong et al. (122) had previously demonstrated presynaptic effects of PregS. Lee et al. (121) identified a role for PregS in eliciting presynaptic plasticity by altering intracellular Ca^2+^ via Ca^2+^-induced Ca^2+^ release (CICR). Moreover, PregS modulates CICR, which is a key mediator of neuronal plasticity. PregS may affect CICR indirectly by activation of NMDARs or L-type voltage-gated Ca^2+^ channels (L-Type VGCCs) and not by direct activation of the Ca^2+^ release-activated Ca^2+^ channel protein 1 (ORAI1) or stromal interaction molecule 1 (STIM1).

DHEA, which is structurally similar to PREG, is the most abundantly expressed neurosteroid in the human body. This neurosteroid is synthesized in the brain, and higher concentrations are found in the brain than in plasma (123). DHEA and DHEAS are neuroprotective, acting via NMDA and AMPA receptors (124, 125). DHEA also appears to play a role in neuronal cell differentiation and programmed cell death via interactions with neurotrophic tyrosine kinase receptors (126, 127). DHEAS, which is structurally similar to PregS except for substitution of carbonyl oxygen for the acetyl group at C17 on the steroid D ring, potentiates NMDA-mediated Ca^2+^ currents and inhibits GABA_A_R-mediated chloride currents (124, 128–131). Neurosteroids are neuroprotective and reduce neuroinflammation (124, 125, 132, 133).

### The Modulatory Recognition Sites

There is a paucity of information on the direct binding of neurosteroids to receptors and of the mechanisms underpinning neuromodulation (39, 134). The ability of neurosteroids to bind to and activate specific categories and subtypes of neuronal receptors is influenced by: (1) conjugation of the parent molecule with a sulfate group; (2) geometry (planar vs. bent); and (3) charge (38, 119). The complexity of neurosteroid-mediated effects, for instance gating of GABA_A_R (109, 135–139) and subunit-specific modulation of glycine and NMDARs (140), suggest the likelihood of multiple binding sites that contribute to potentiating and inhibitory effects (39). The effects of neurosteroids on GABA_A_Rs also appears to involve modulation of δ subunit-containing receptors which play a role in tonic inhibition (141–145).

**Figure 3:**
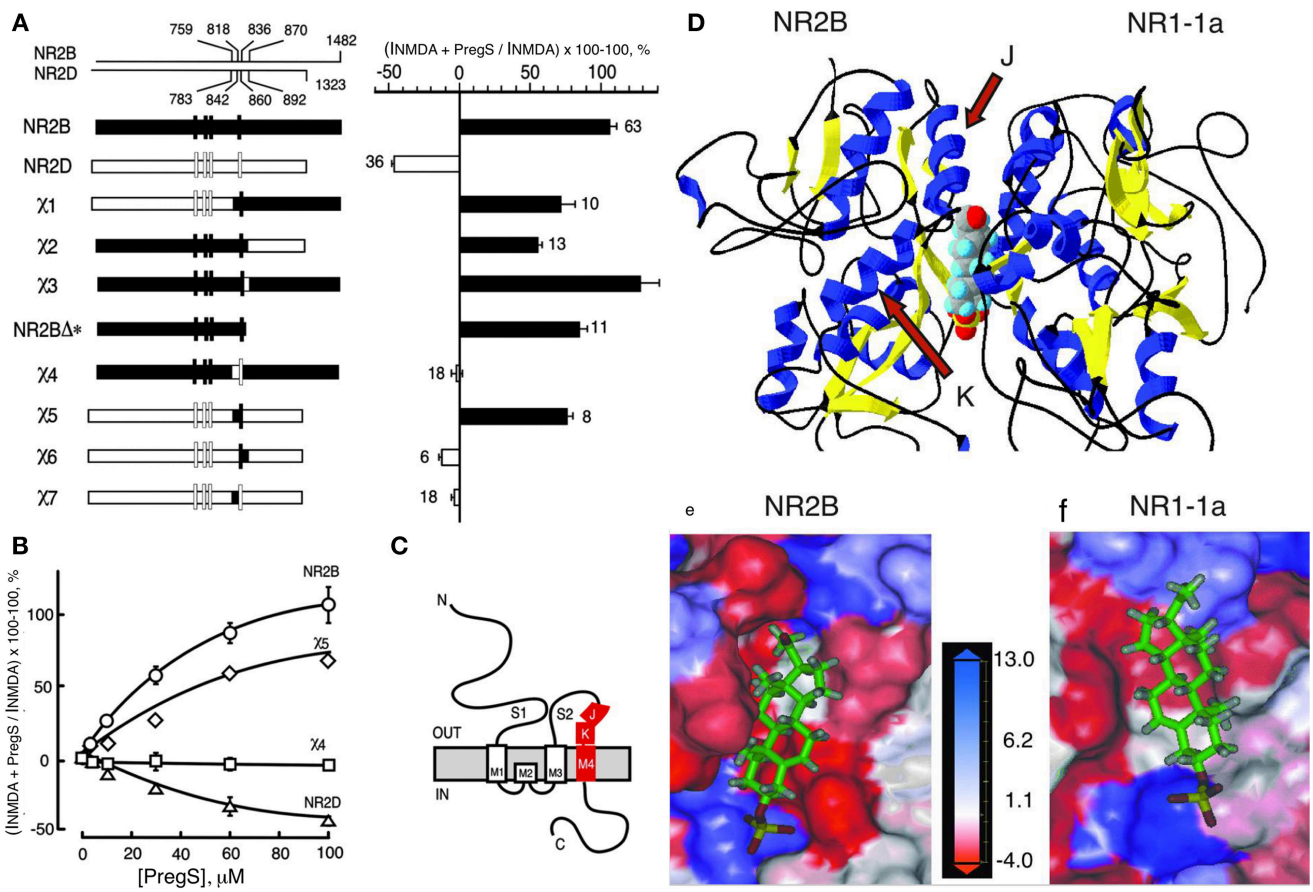
Steroid modulatory domain of NMDARs. **(A)** (Left) Schematic representation of wildtype NR2B, NR2D, and the NR2B, NR2D chimeras. The contribution of NR2B and NR2D to chimeras is depicted in black and white, respectively. The scales at the top indicate the residue numbers in the wildtype subunits at junctions. Vertical bars represent the four hydrophobic membrane domains. (Right) Percent increase in the NMDA glycine response (elicited by 300 μM NMDA and 50 μM glycine in oocytes expressing NR1-1a and NR2 subunits) in the presence of 100 μM PS is indicated. Error bars are SEMs. Numbers adjacent to the error bars indicate the number of oocytes used in the study. **(B)** Concentration–response curves of PregS modulation for receptors containing NR2B (○), NR2D (Δ), χ4 (), and χ5 (♢), were determined in the presence of saturating concentrations of NMDA (300 μM) and glycine (50 μM). The EC50 (NMDA) for NR1-1a χ4 and NR1-1a_NR2B are both 22 ± 1 μM, and EC50 (glycine) is 0.30 ± 0.02 and 0.10 ± 0.02 μM, respectively. **(C)** The topological representation of the NR2B subunit and the location of the identified segment are depicted in red. Membrane domains are denoted as M1–M4. The amino terminus (N) is located on the extracellular side and the carboxyl terminus **(C)** on the intracellular side of the plasma membrane. **(D)** Molecular modeling of potential binding pocket for PregS. The dimer comprising the S1/S2 domains of NR2B and NR1-1a is depicted in a 3D ribbon structure with helices colored in blue and sheets colored in yellow with PregS docked at the interface between the two subunits. Our finding that both J and K helices (see arrows) and M4 of the NR2B subunit are required to confer PS potentiation indicates that M4 is also critical in coupling allosteric modulation from extracellular binding regions to the gating mechanism. **(E)** Detailed view of the potential binding pocket for PregS on NR2B. **(F)** Detailed view of the potential binding pocket for PregS on NR1-1a. NR1-1a or NR2B have been removed from the models to show the hydrophobic pocket on NR2B **(E)** or NR1-1a **(F)**, respectively. The receptor surface is colored according to a hydrophobicity scale with hydrophobic residues in red and charged residues in blue. PregS is depicted in a stick configuration and colored by the atom type with hydrogen in white, carbon in green, oxygen in red, and sulfur in yellow. Our finding that both J and K helices (see arrows) and M4 of the NR2B subunit are required to confer PS potentiation indicates that M4 is also critical in coupling allosteric modulation from extracellular binding regions to the gating mechanism [From Jang et al. (43) with Permission].

**Figure 4:**
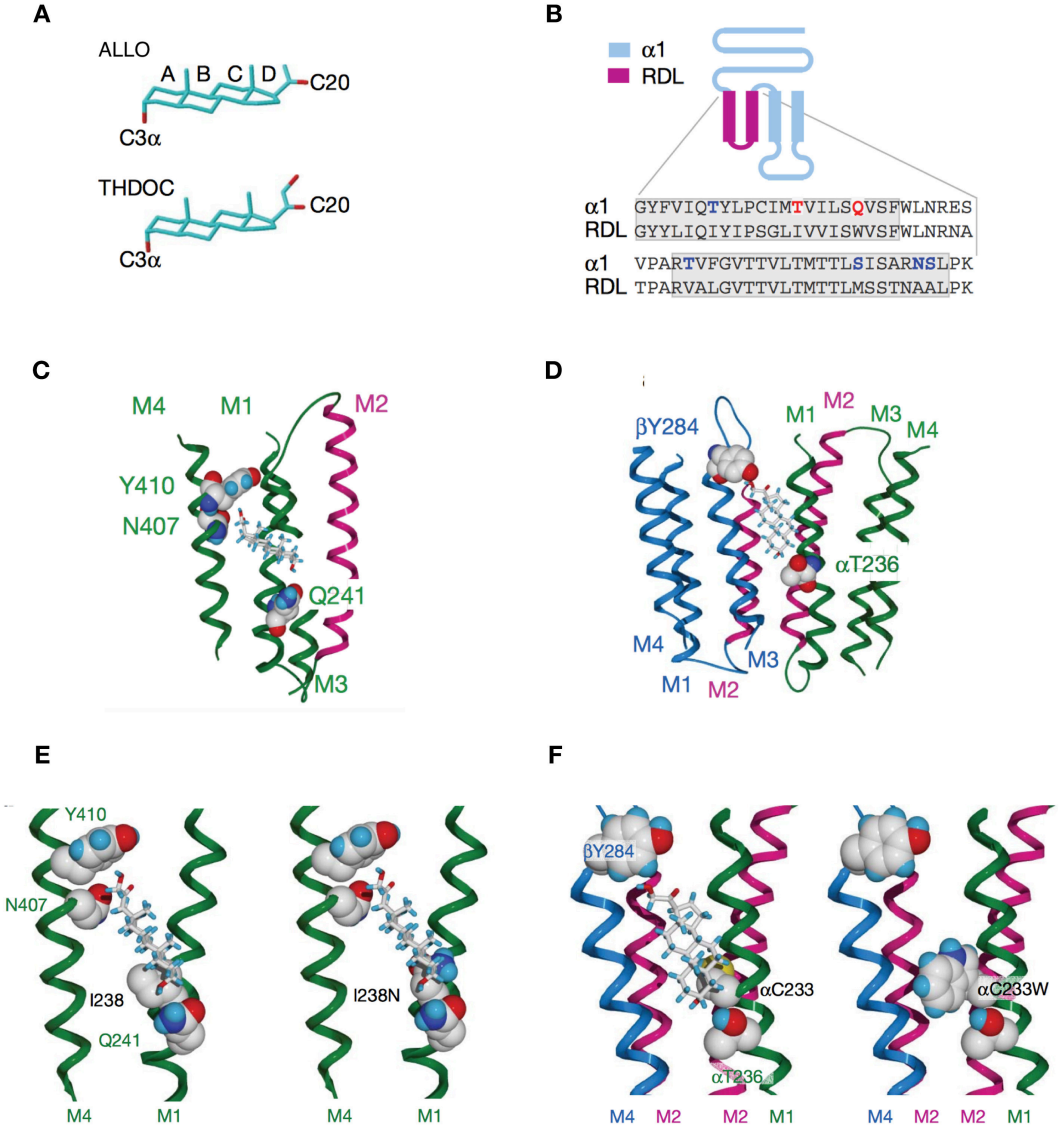
Activation of GABA_A_Rs by neurosteroids, such as ALLO and THDOC depends on occupancy of both the activation and potentiation sites on the transmembrane domain. **(A)** Regulation of GABA_A_R, is dependent on the C3a hydroxyl group on the A-ring and the C20 ketone on the D-ring shown here in structures of ALLO and THDOC. **(B)** *Neurosteroid activity is determined by* α*-subunit M1 domain residues*. Replacement of membrane domains M1 through to the end of M2 in the murine α1 and β2 subunits with the corresponding sequence from the RDL subunit, forming the chimeras αR and βR, respectively, was the first modification used to established their GABA_A_R pharmacology. Potentiation and direct activation of GABA_A_Rs by THDOC and ALLO was abolished in chimeric receptors incorporating αR; receptors containing βR were indistinguishable from the wild type. Polar residues in α1 (blue) are in bold, with Thr 236 and Gln 241 (red) highlighted. The transmembrane domains are boxed. **(C)** *Neurosteroid potentiation requires* α*-subunit M1 and M4 membrane domains*. Ribbon structure of α subunit viewed from the lipid bilayer showing αGln 241, αAsn 407, and αTyr 410 docking with a THDOC molecule. The channel lining the M2 membrane domain is shown in purple (a section of M3 domain is omitted for clarity). **(D)** *Neurosteroid activation binding site spans the* β*/*α*-subunit interface*. View of transmembrane region (extracellular and cytoplasmic domains removed) with a bound THDOC molecule. Replacing αThr 236 with non-hydrogen-bonding isoleucine or valine reduces the agonist potency of ALLO and THDOC. **(E)** *Neurosteroid potentiation requires* α*-subunit M1 and M4 membrane domains*. Homology model of THDOC bound to the potentiation site between M1 and M4 membrane domains of the [David: should the following show alpha or beta symbol?] α subunit (M3 membrane domain removed from figure for clarity). Ile 238 is predicted to lie close to the A-ring of THDOC (left). Introduction of a similar-sized but polar side chain at residue 238, such as replacement with asparagine repels the steroid (right). **(F)** Replacement of Cys 233 (left) with tryptophan (right) increases the steric hindrance for THDOC binding to βTyr 284 and αThr 236 [From Figures 1, 3, 4 of Hosie et al. (109)].

**Figure 5:**
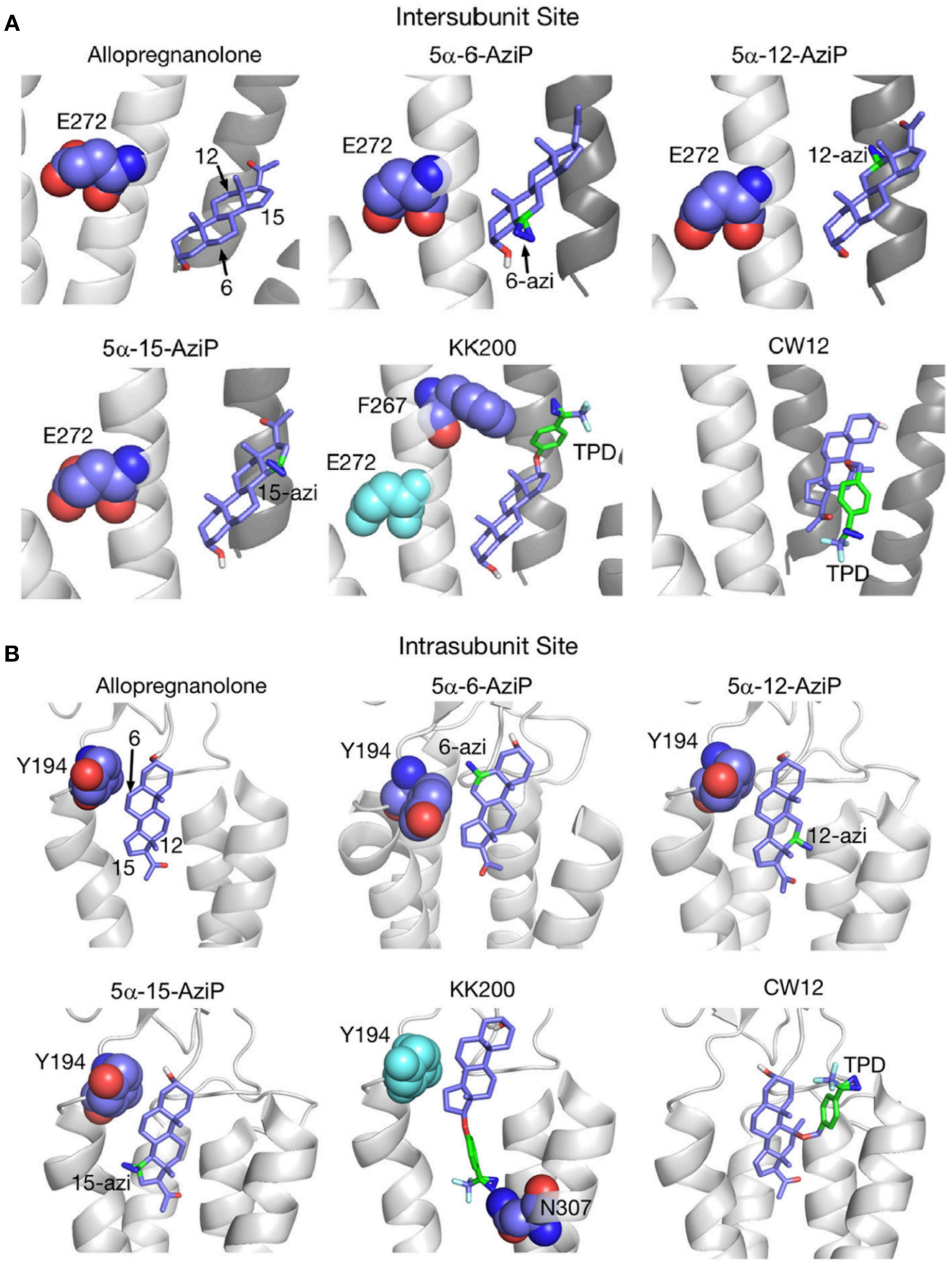
Docking positions of ALLO and photolabeling reagents in the intersubunit and intrasubunit sites. **(A)** Intersubunit docking sites; the photolabeled residues for Glu-272 (5α-6-AziP, 5α-12-AziP, 5α-15-AziP) and Phe-267 (KK200), are shown as purple spheres. **(B)** The intrasubunit docking sites; the photolabeled residues, Tyr-194 (5α-6-AziP) and Asn-307 (KK200), are shown as purple spheres [From Cheng et al. (134), with Permission].

The receptor transmembrane domain plays a role in neurosteroid-mediated modulation of NMDARs (43) (Figure 3) and GABA_A_Rs (109, 146, 147). Residues in the α1-subunit M1 and/or M2 membrane domains of the GABA_A_Rs are critical for neurosteroid action (109) (Figure 4) Recent studies using Gloeobacter ligand-gated ion channels (GLIC), a prototypic pentameric ligand-gated ion channel that is a homolog to the nicotinic acetylcholine receptor, have identified putative intersubunit and intrasubunit neurosteroid binding sites for ALLO within the transmembrane domain (134) (Figure 5). Using this innovative approach, Cheng et al. (134) found that substitutions at the 12 and 15 positions on the neurosteroid backbone altered modulation of GLIC channel activity, demonstrating the functional role of both sites. The interaction of neurosteroids with GABA_A_R is stereoselective, suggesting that the binding sites for these compounds are of a specific dimension and shape (12, 34, 148).

The results suggest that interactions between the extracellular domain and transmembrane domains play an essential role in the positive and negative modulatory actions of neurosteroids (149). Another important factor determining neurosteroid-dependent modulation of cell function is localization of the neurosteroid. Recent studies have started investigating this aspect using both endogenous neurosteroids and their synthetic analogs. Neurosteroids that can permeate cell membranes can be localized within different intracellular compartments. This compartmentalization is likely to be of importance to the therapeutic function of the neurosteroid (150).

Other known molecular targets of neurosteroids include various TRP channels (151, 152), serotonin receptors and L-Type VGCCs (42). Subtypes of the TRP channels expressed in mammals include TRPC (canonical), TRPV (vallinoid), TRPM (melastatin), and TRPA (ankyrin) channels. PregS modulates Ca^2+^ influx via TRPM3 channels in pancreatic beta cells (151). In addition, TRPM3 is also activated by related substances PREG, DHEA and DHEA sulfate in these studies. Although previous studies by Chen and Wu (153) suggest that PregS activates TRPV1, also known as the capsaicin receptor, other members of the TRPV and TRPM subfamily tested by Wagner and colleagues were not activated by PregS.

**Figure 6:**
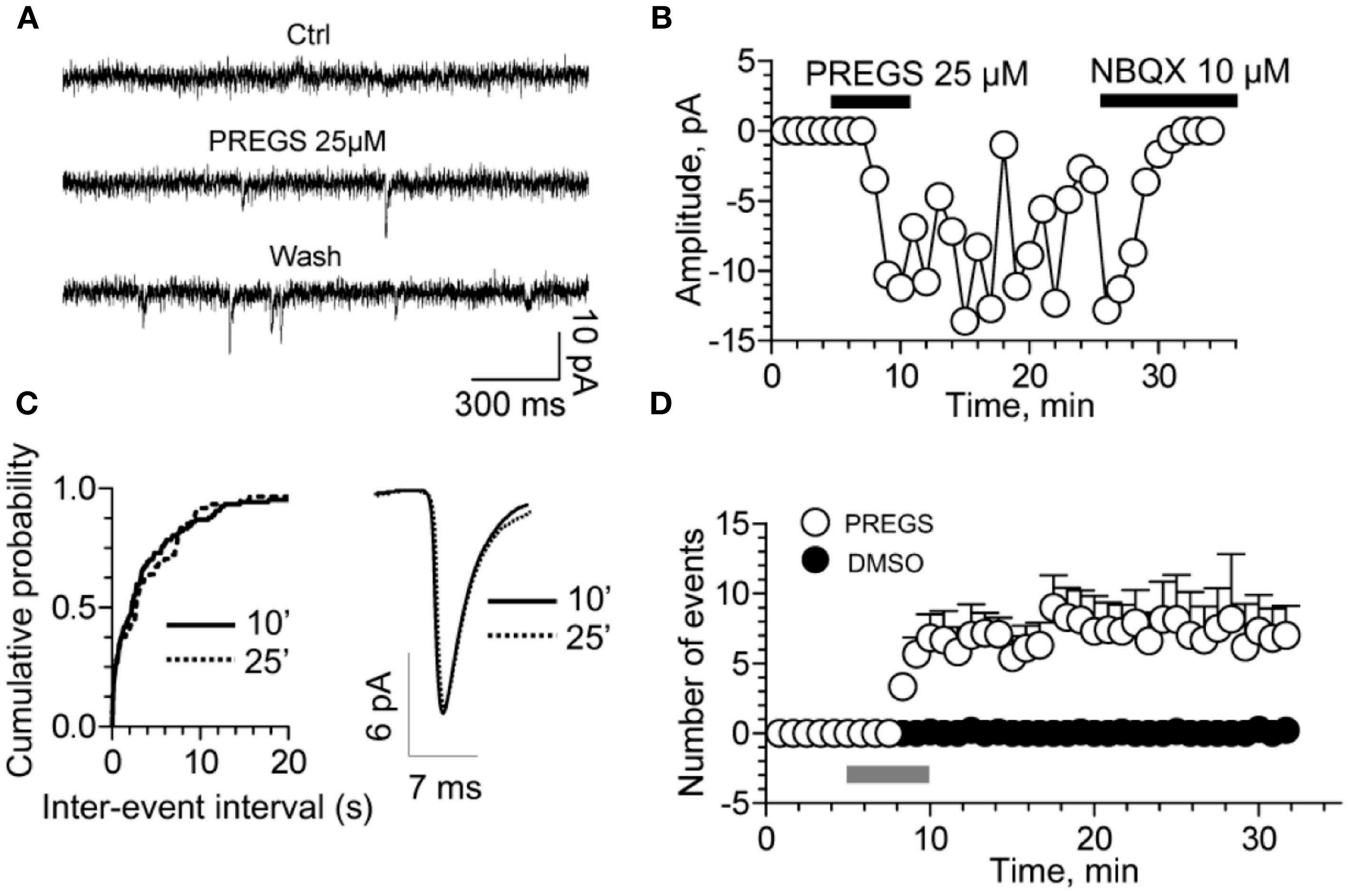
PregS activates silent synapses. **(A)** Sample traces of whole-cell patch-clamp recording from CA1 pyramidal neurons in hippocampal slices from a P3 rat reveal mEPSCs after 25 μM PregS exposure. This effect, which is absent under control conditions, is not reversed by washout. **(B)** Depiction of the time course for the recordings shown in figure **(A)**. Note that NBQX blocks this effect, indicating it is mediated by AMPA receptors. **(C)** Cumulative probability plot. The distribution of the mEPSC inter-event intervals at 10 vs. 25 min is not influenced by PregS exposure. Average mEPSC traces illustrating the lack of an effect of PregS on mEPSC amplitude at these time points. **(D)** Summary figure showing effects of vehicle (DMSO) and PregS (application represented by the gray bar) on the number of events as a function of time (*n* = 8–12) [From Valenzuela et al. (156) with Permission].

**Figure 7:**
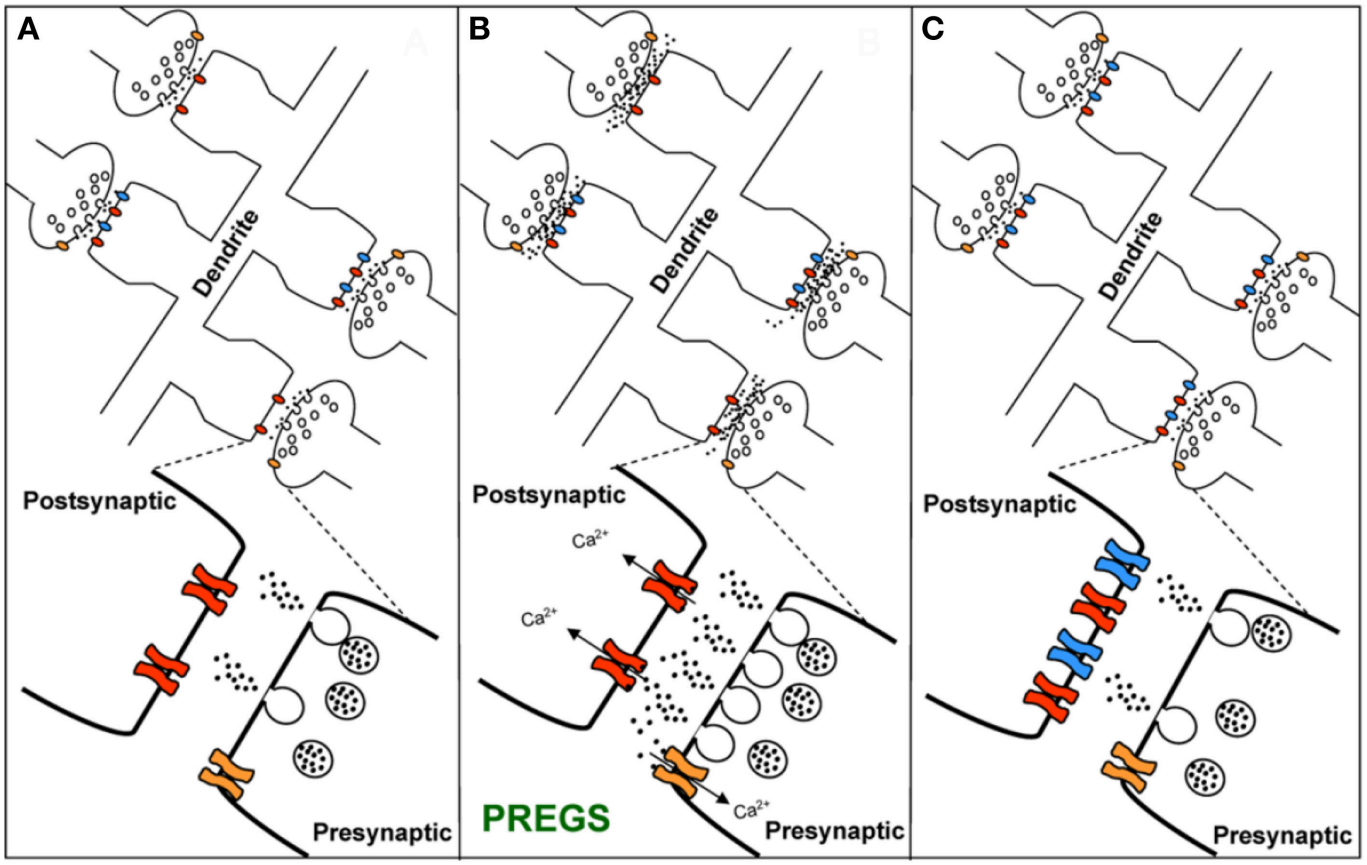
Working model for the action of PregS and related neurosteroids at glutamatergic synapses in immature CA1 hippocampal pyramidal cells. **(A)** Under control conditions, neonatal CA1 hippocampal pyramidal neurons could have a mixture of glutamatergic synapses containing both post-synaptic AMPARs (blue) and NMDARs (red) (active synapses) or only post-synaptic NMDARs (silent synapses at rest). A “*silent synapse*” is enlarged. Presynaptic NMDARs are shown in orange. **(B)** PregS increases Ca^2+^ influx through presynaptic NMDARs, leading to an increase in the probability of glutamate release and activation of post-synaptic NMDARs. Pre- and post-synaptic NMDARs at these synapses are proposed to contain NR2D subunits, making them less sensitive to Mg^2+^ block. **(C)** Glutamate release probability returns to baseline levels after PregS washout. The post-synaptic increase in [Ca^2+^] shown in panel B elicits delayed insertion of AMPARs only in “*silent synapses*” [From Valenzuela et al. (156) with Permission].

**Figure 8:**
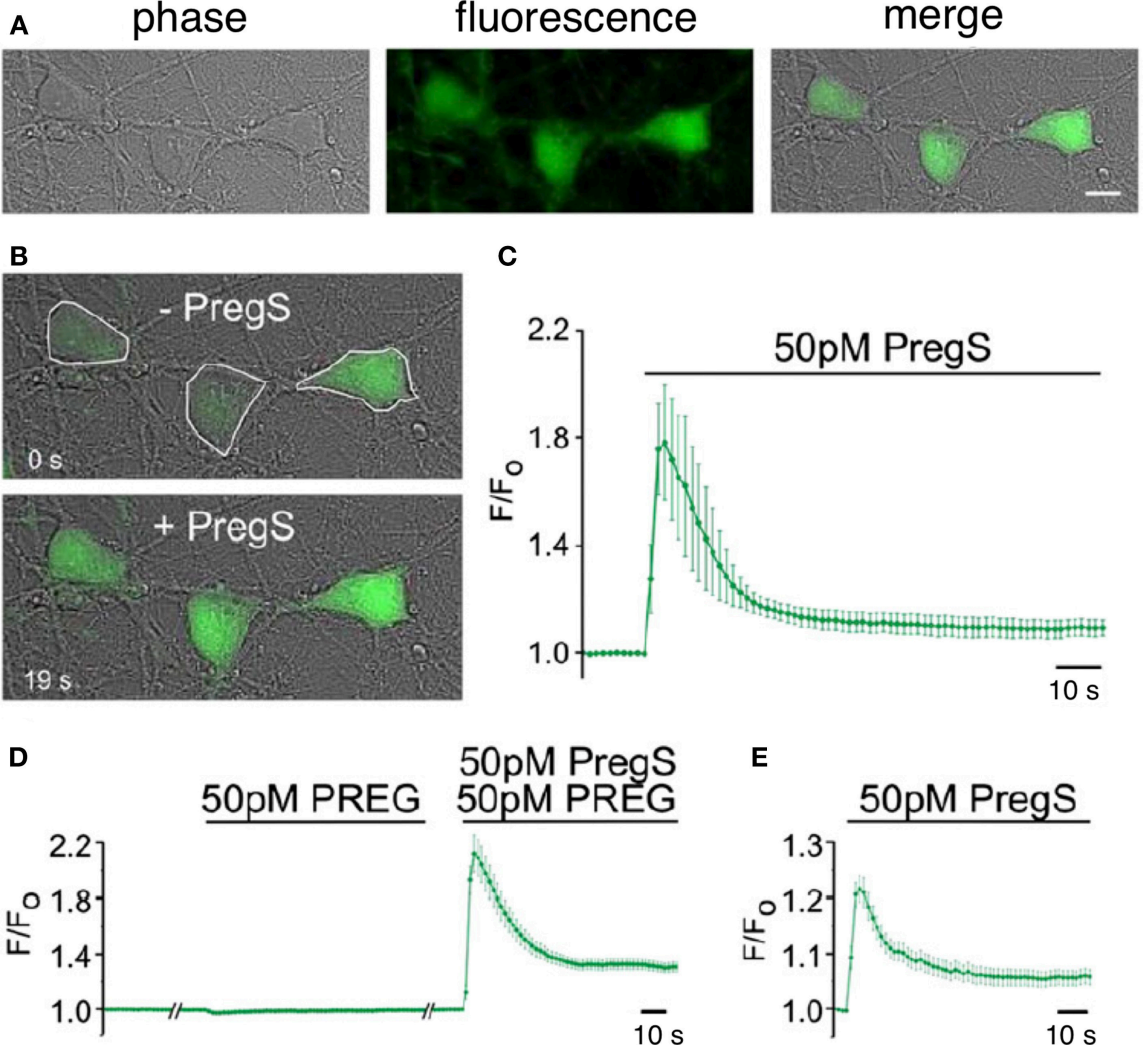
Picomolar concentrations of PregS increase [Ca^2+^]_i_ in primary cultured cortical neurons. **(A)** Phase-contrast (left), fluorescence (middle), and merged (right) image of a field of cortical neurons. **(B)** Before (top, 0 s) and after (bottom, 19 s) application of 50 pM PregS. Regions of interest defined morphologically as neuronal cell bodies are outlined in white. **(C)** Mean 6 S.E.M. fluorescence intensity normalized to average initial intensity of the same cell (F/Fo) for 3 neurons in **(B)**. **(D)** Baseline Ca^2+^ fluorescence is shown in the (left) trace. PREG (50 pM) does not increase [Ca^2+^]I (middle trace), whereas a subsequent application of 50 pM PregS in the presence of 50 pM PREG increases [Ca^2+^]i. (right trace) (mean 6 S.E.M., 30 neurons). **(E)** 50 pM PregS increases [Ca^2+^]i in primary cultured hippocampal neurons. Scale bar for **(A,B)**: 10 mm. [From Smith et al. (48) with Permission].

**Figure 9:**
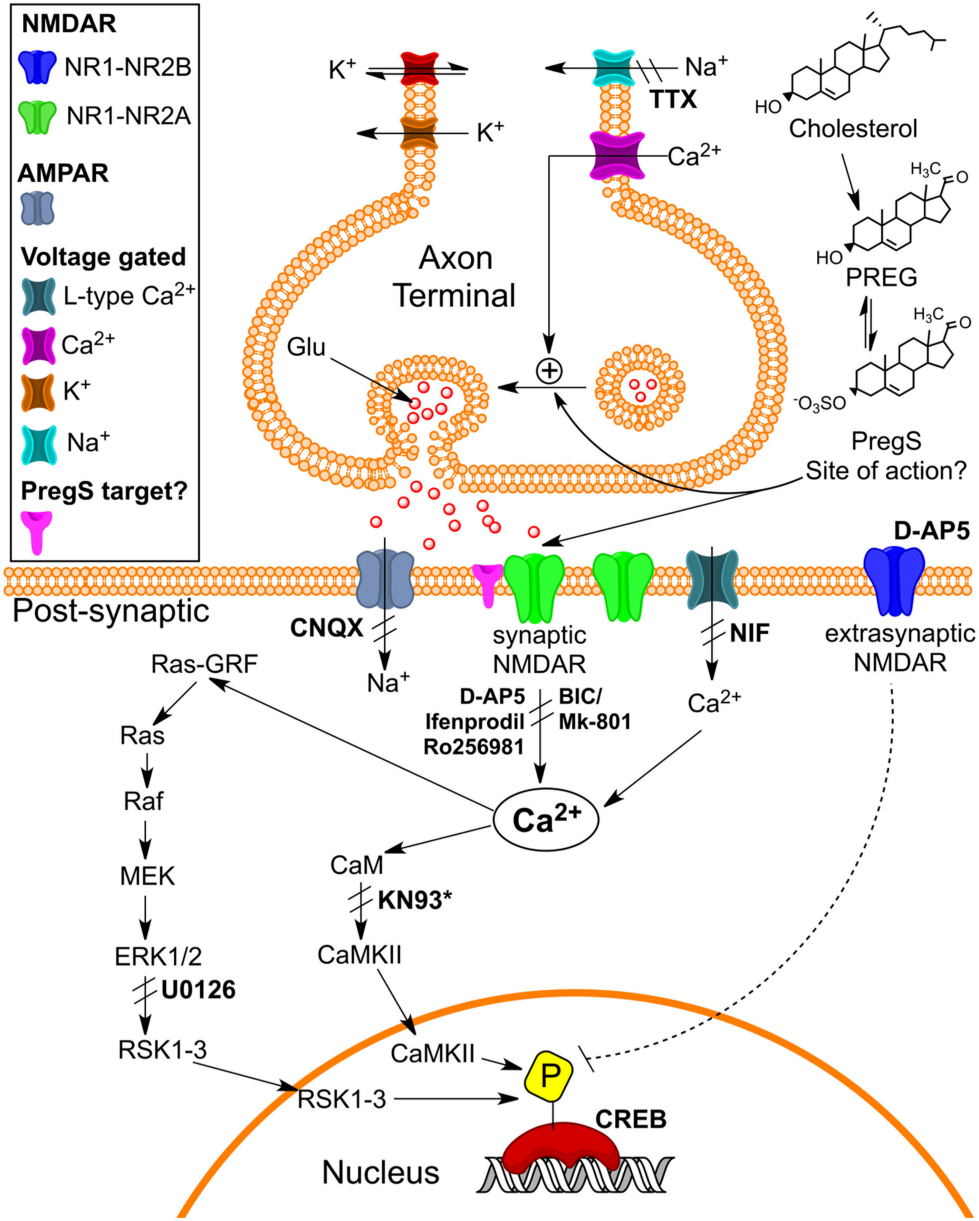
Schematic illustrating pathways that may underlie pM PregS-induced [Ca^2+^]i and pCREB increases. Diagram illustrates 50 pM PregS-stimulated increase in [Ca^2+^]i via voltage-gated Na+ channels, NMDARs, and Ca^2+^L and 50 pM PregS-induced pCREB increases via synaptic NMDAR and ERK activation. Inhibitors used in the study are in bold. ^*^KN93 did not inhibit PregS-induced pCREB increases. [From Smith et al. (48) with Permission].

TRPM3 channels are expressed at glutamatergic synapses in neonatal Purkinje cells (154). The effect of PregS on AMPA receptor-mediated miniature excitatory post-synaptic current (mEPSC) frequency is blocked by lanthanide^3+^, a non-selective TRP channel blocker (155), providing support for TRPM3 as a target for PregS modulation of glutamate release. Valenzuela et al. (156) demonstrated that PregS activates silent synapses by promoting Ca^2+^ influx in a TRP channel-dependent manner, increasing presynaptic glutamate release, and insertion of AMPARs into the post-synaptic membrane (Figure 6). Based on these results, Valenzuela et al. developed a working model to explain the actions of PregS at glutamatergic synapses (Figure 7). This model is consistent with the recent discovery of delayed onset potentiation of the NMDA response reported by Kostakis et al. (47). We subsequently demonstrated that the phenomenon of delayed onset potentiation of the NMDAR response induced by PregS occurs at physiologically relevant picomolar concentrations and is coupled to a downstream signal transduction pathway associated with learning and memory function (Figures 8, 9).

Modulation of TRP channels has been proposed as a therapeutic target for age-related neurologic disorders, such as AD (157). TRPA1 channels regulate astrocyte resting Ca^2+^ and inhibitory synapse efficacy (158). The TRPA1 channel has also been implicated in astrocytic hyperactivity and synaptic dysfunction mediated by Aβ in mouse models of AD (159). Recognized differences in TRPA1 channels across species have been cited as confounding the translational value of results from preclinical rodent models (160). For example, human TRPA1 activity is suppressed by caffeine but mouse TRPA1 channels are activated (161). More work is needed to fully elucidate the role of neurosteroids as functional modulators of the different subtypes of TRPs expressed in the brain.

### Transport of Negatively Charged Steroids Across Cell Membranes

Sulfated steroids, namely PregS and DHEAS, are unique in that they are highly negatively charged and thus do not pass across cell membranes without specific transporters. It therefore seems unlikely, although not impossible, that PregS or DHEAS could rapidly associate with a receptor present within the cell without a specific membrane transporter. Our results show that PregS acts extracellularly (35) and that PREG is inactive as a modulator (36–38, 162). For these reasons, PregS more closely resembles the characteristics of classical neurotransmitters, such as acetylcholine, in which the parent molecule bears a full positive charge and is active only at an extracellularly directed recognition site while the immediate breakdown products (choline + acetic acid) are inactive. However, with respect to the neuronal membrane, PregS exhibits full effect even when applied extracellularly to cultured cortical and hippocampal neurons (27, 48) internally dialyzed with the same concentration of PregS via the whole cell patch clamp recording configuration (47).

The recognition site for steroids, such as estrogen (ER), progesterone and its metabolite ALLO is now relatively well-described. For instance, membrane-bound ER receptors, such as ERα localize at the cell surface where they regulate cell signaling mediated by ER produced in the CNS; this function is in addition to their well-recognized classical intracellular localizations where these receptors function to modulate transcription (163–166). Interestingly, activation of these membrane receptors leads to rapid, non-genomic effects and regulates neuronal plasticity in the CNS (166–171).

An extracellular site of activation for PregS on endogenous NMDARs in primary rat hippocampal and chick spinal cord neurons in culture, as well as receptors expressed in *Xenopus* oocytes, was demonstrated by Farb et al. (26). Extracellular application of PregS activates NMDARs, whereas intracellular dialysis with PregS fails to elicit a neuronal response or to inhibit PregS applied extracellularly (35). An extracellularly directed PregS -binding domain and obligatory transmembrane domain TM4 participate in the positive allosteric modulation of NMDAR activation by neurotransmitter co-agonists glutamate and glycine [(43, 44) and reviewed by (62, 172)].

These findings were recently confirmed and extended by Wilding et al. (54), who generated chimeric receptors by replacing specific domains of the NMDAR with homologous domains from kainate receptors expressed in non-neuronal HEK293 cells to elucidate the contribution of specific domains to pore formation and allosteric modulation of the NMDARs. By contrast, potentiation by the dihydroxysterol does not require the ligand-binding domain but instead requires a membrane proximal portion of the carboxy terminal domain of the NMDAR, consistent with an intracellularly directed site for receptor activation. Recently, Chisari et al. (58) have described the characterization of compound analogs of PregS, such as KK169. Like PregS, KK169 has the ability to potentiate NMDAR function, and possesses several characteristics that are consistent with an action for PregS and related sulfated steroids at a cell surface-oriented activation domain (35, 43). Interestingly, KK169 does not inhibit oxysterol potentiation of the NMDAR, consistent with its action at an extracellularly directed binding site (similar to that for PregS). However, some sequestration of KK169 was observed in cultured hippocampal neurons, revealing a possible mechanism for membrane transport and accumulation.

These considerations also relate to question of whether PregS might be able to cross the BBB, with the clear expectation that a specific transmembrane transporter would be needed. In fact, such a transporter has been observed in non-fenestrated intracerebral capillaries (173–177) and could well be present in glia and/or neurons, providing a possible pathway toward sequestration.

## Neurosteroids and Memory Function

PREG was initially thought of only as a precursor for other steroids and not as an active modulator. Fluorescence spectroscopy studies of the binding of PREG and the related sulfated neurosteroid 3α-hydroxy-5β-pregnan-20-one sulfate, which differentially modulate NMDA and AMPA receptors, suggest that the differential effects of these sulfated neurosteroids on current flow may be related to their binding at the SIS2 and amino terminal domains of these receptors (39). Cannabinoid receptor 1 (CB1) has been identified as a molecular target for PREG (178). Tetrahydrocannabinol or THC, the active ingredient in cannabis, induces PREG synthesis in a CB1 receptor-dependent manner. PREG then acts as an allosteric negative modulator of CB1 receptor in an autocrine-paracrine loop in the brain acting to ameliorate cannabis intoxication. CB1 receptor activation is well-known to modulate learning and memory function by depressing neurotransmitter release. Interestingly, two cannabinoid receptor-mediated signaling cascades have been identified: one is PREG sensitive and targets the vesicular protein Munc-18-1, thereby depressing transmitter release; the other is PREG-insensitive and involves the lateral perforant path of the hippocampus (179). When given as an adjunctive treatment to patients diagnosed with schizophrenia, PREG both improves negative symptoms and ameliorates cognitive deficits (50).

**Figure 10:**
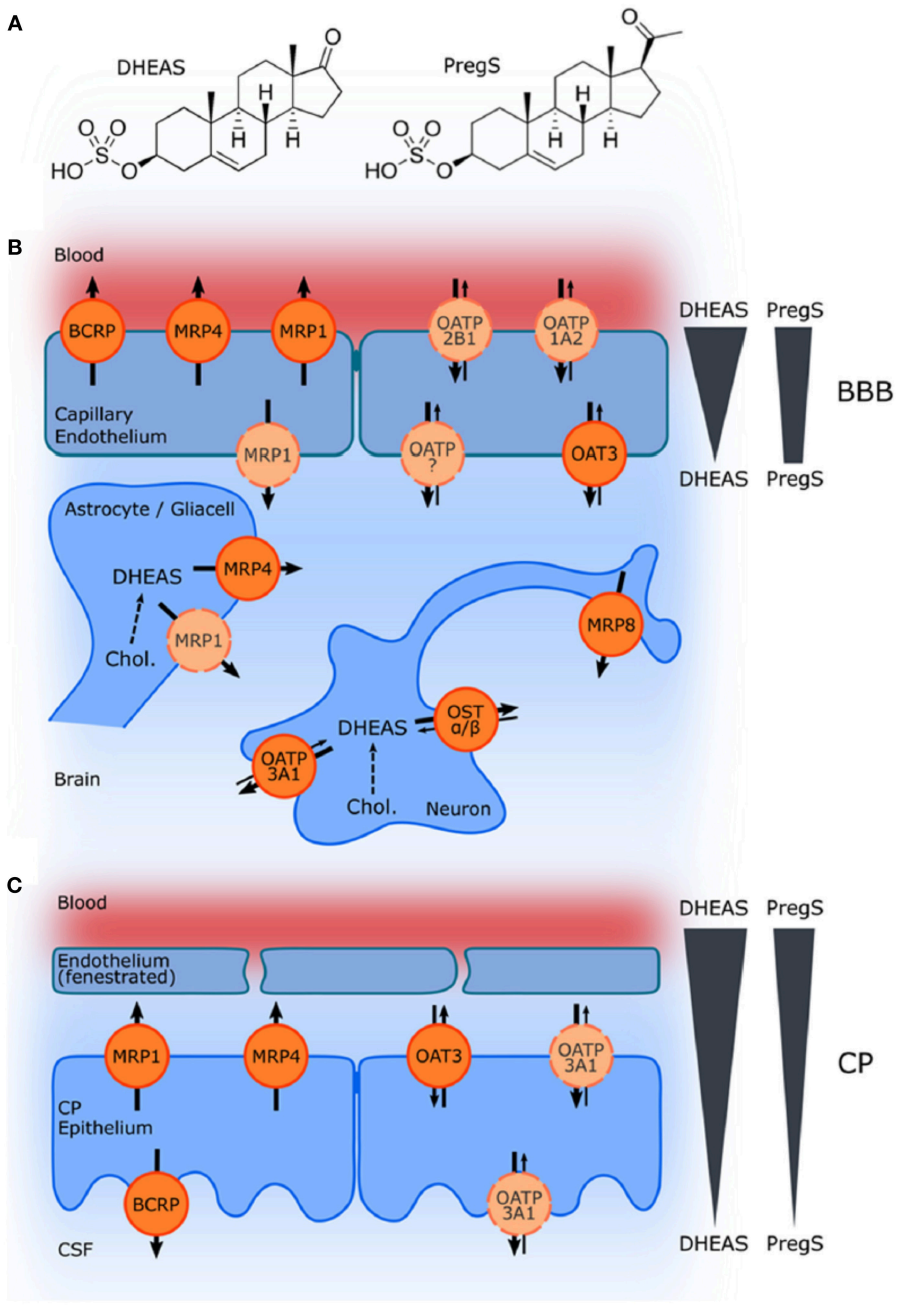
Schematic illustration of ABC and SLC transporters putatively involved in DHEAS and PregS transport and their proposed localization. **(A)** Structures of DHEAS and PregS. **(B,C)** The ATP-binding cassette (ABC) and solute carrier (SLC)-type membrane proteins facilitate transport of neurosteroids at the BBB and in the choroid plexus (CP) The ABC transporters BCRP (ABCG2), MRP1, MRP4, and MRP8 (ABCC1, ABCC4, and ABCC11) facilitate efflux of conjugated steroids. The solute carriers OAT3 (SLC22A3), OATP1A2 (SLCO1A2), OATP2B1 (SLCO2B1), and OSTa/ß (SLC51A/B) are implicated in secretion of sulfated steroids from neurons and glial cells and in their transport across the BBB as well as blood–CSF barrier in the CP. Arrows indicate the directions of substrate transport. Proteins for which there is little or controversial evidence for expression and localization in the basal or apical membrane are indicated in light orange and by a dashed line. The DHEAS and PregS concentration gradients across the BBB and in the CP are depicted on the right [From Grube et al. (177) with Permission].

Although controversy still remains with respect to the ability of systemically administered sulfated neurosteroids to cross the BBB, acute treatment with PregS, which is well-recognized for its actions as a positive allosteric modulator of NMDARs, has been associated with an improvement in learning and memory function (21, 24, 31, 34, 35, 162, 180–185). The influx of the sulfated compounds is dependent upon transporters, such as organic anion transporting peptides (OATPs) situated in the BBB and choroid plexus (173, 177, 186) (Figure 10).

The role of these transporters in the efflux of negatively charged sulfate steroids across the BBB from the CNS into the systemic circulation is fairly well-established; however, the specific mechanisms associated with brain influx have not been fully elucidated, despite the finding that systemic administration of sulfated steroids produces effects on cognition including improved learning and memory function (31, 173, 176). It had been suggested, based on studies looking at the expression of 17α-Hydroxylase/C17-20-lyase and hydroxysteroid sulfotransferase, that sulfated steroids, such as DHEAS are unlikely to be synthesized *de novo* within the human hippocampus (186). This work has led to the suggestion that sulfated neurosteroids must be transported from the periphery into the CNS, but this hypothesis has not been validated (186). Using *in situ* rat brain perfusion, Qaiser et al. (176) found that PregS enters the brain more rapidly than DHEAS and that both sulfated steroids undergo extensive desulfation mediated by sulfatase located in the capillary fraction of the BBB. While more work is clearly needed to parse out these complex relationships (95), systemic administration of conjugated neurosteroids can nevertheless result in increased CNS levels of the unsulfated neurosteroids (176).

Whether or not the cognitive enhancing effects are due to PregS or PREG remains to be determined. However, the cognitive enhancing effects of PregS in healthy subjects are unlikely to be due to the PREG metabolite ALLO since acute administration of this and other GABAergic modulating neurosteroids to healthy subjects inhibits learning and memory function in a manner similar to benzodiazepines (187–191). On the contrary, ALLO may play a role in the effects of PREG seen in patients with schizophrenia and other neuropsychiatric and neurologic disorders in which neural network activity is dysregulated (51).

Unlike synthetic pharmaceuticals, the literature reporting on the role of neurosteroids in learning and memory function includes some studies looking at the role of endogenous levels and other studies looking at the effects of systemically administered neurosteroids. Because of this, there are several important factors that must be considered when interpreting the results of these studies, including: (1) metabolism of the parent neurosteroid molecule and active metabolites may have a different mechanisms of action; (2) dosing schedules can be acute or chronic; and (3) pharmacokinetic and pharmacodynamic interactions between the neurosteroids and endogenous levels of circulating steroid hormones can fluctuate over time.

Performance on tests of memory function also depends on when neurosteroids are administered in relationship to training or testing. For example, administration of neurosteroids before training can influence both acquisition and consolidation of new information, whereas administration after training is expected to influence consolidation and recall but not acquisition. The type of memory function being assessed (e.g., working memory vs. long-term memory) may also be more or less sensitive to the effects of neurosteroids. Finally, the age and cognitive status of the study subjects must be considered. Each of these factors is explored in greater detail below.

### Mechanism of Action and Metabolism of Parent Molecule to Active Metabolites

Neurosteroids are metabolized to other neurosteroids that can also influence neurologic function. For example, PREG is a lipophilic precursor of ALLO, which is also a metabolite of progesterone. Administration of a loading dose of PREG is associated with an increase in serum ALLO levels (192). Although PREG is preferentially metabolized to ALLO, it is also metabolized to progesterone and DHEA (51, 52, 104). The extent to which a neurosteroid, such as PREG is metabolized to progesterone and vice versa determines whether the dose administered will modulate extra- or intracellular receptors to produce non-genomic vs. genomic effects (104).

Administration of 5α-reductase inhibitors, such as finasteride appears to prevent the metabolism of progesterone to ALLO and may thereby influence the neuromodulatory effects of endogenous as well as exogenous sources of these neurosteroids (193–195). Studies looking at the direct infusion of PregS into the nucleus basalis show that this neurosteroid improves spatial memory function in rats and increases acetylcholine release in the basolateral amygdala and frontoparietal cortex (196). Despite these promnestic effect, the metabolism of sulfated neurosteroids, such as PregS by sulfatases, coupled with limited transport across the BBB, has impeded the development of this compound as a novel therapeutic. This limitation can be overcome by using synthetic analogs (132).

### Acute vs. Chronic Dosing Schedules

Acute administration of ALLO improves memory function in one mouse model of AD (197), but other studies using rodent models suggest that chronic exposure to ALLO may actually cause memory deficits and exacerbate disease-related functional impairments (198–200). The observation that ALLO transiently increases CREB phosphorylation and increases indicators of neurogenesis in wildtype rats (201) suggests that the acute and chronic effects may not be the same. The translational relevance of these observations to human subjects is unclear since, although men show an age-related decrease in serum ALLO, a similar age-dependent reduction in circulating levels of this neurosteroid is not seen in women (202). On the contrary, women show changes in circulating levels of ALLO that correlate with transient changes in circulating levels of steroid hormones (202). Interestingly, acute systemic administration of ALLO appears to interfere with episodic memory function in healthy women without impairing semantic or working memory function (203), suggesting that brain regions involved in these distinct memory processes, such as the frontal lobes and hippocampus are differentially modulated by this neurosteroid.

Studies looking at brain region-specific effects of neurosteroids on memory function have served to further elucidate the role these neuromodulators play in the acquisition, consolidation and retrieval of information. Systemic administration of the GABA_A_R positive modulator ALLO to mice interferes with acquisition and consolidation on a hippocampal-dependent novel object recognition task (204, 205). ALLO impairs memory acquisition/encoding in rodent models when it is injected into the nucleus basalis magnocellularis *before* an acquisition trial, implicating inhibition of cholinergic neurotransmission in the effects of ALLO on memory acquisition (181). Intrahippocampal administration of ALLO into the CA1 subregion *after* the acquisition phase of a passive avoidance paradigm has no measurable effect on retention (206).

### Age-Dependent Effects of Neurosteroids

Neuroactive steroid hormones levels change during development, with aging and across the estrous cycle (207, 208). Age-dependent effects of neurosteroids on memory function are seen in rodents and humans. For example, neonatal exposure of female rats to estradiol has been associated with reduced brain levels of ALLO and improved learning and memory function in adulthood (209). It has been suggested, based largely on work in animal models, that enduring changes in GABA_A_Rs expression induced by developmental exposure to steroid hormones, such as progesterone and its metabolites play a role in hippocampal neuronal excitability and in the etiology of sex-dependent neuropsychiatric disorders and memory deficits later in life (209–211). Administration of the 5α-reductase inhibitor, finasteride to pregnant rat dams late in gestation impairs cognitive and neuroendocrine function in their juvenile offspring (210). Treatment of neonatal female rats with estradiol increases the expression of extrasynaptic α4/δ subunit-containing GABA_A_Rs and improves performance in the Morris water maze during adulthood (209).

In humans, serum levels of ALLO are normally stable during the first 2 years of life (212). Obese children have been reported to have elevated circulating levels of ALLO, but how this influences their learning and memory function later in life has not been elucidated (213). In post-menopausal women, the effects of ALLO on mood appears to be dose dependent and to follow an inverted U-shaped cure (214). The major contributor to endogenous levels of ALLO is the corpus luteum of the ovary and, not too surprisingly, serum levels of ALLO increase during puberty and with polycystic ovary syndrome (PCOS) (202, 212, 215–218). Functional imaging studies indicate that activity within the right superior and inferior parietal lobes is increased during performance of working memory tasks in untreated women with PCOS. The observed increase in neuronal activity is attenuated by antiandrogen therapy (219).

During the menstrual cycle in humans, ALLO and progesterone levels rise together, with the highest concentrations reached in the luteal phase (202, 220–223). Encoding of emotional memories appears to be better in the luteal phase than in follicular phase (207). It has been suggested that cyclical fluctuations in circulating steroid hormone levels can influence encoding and recall of emotional stimuli, and therefore may play a role in the expression of post-traumatic stress disorder (PTSD) symptomology (224, 225).

In rodents, the onset of puberty is associated with an increase in the expression α4βδ GABA_A_Rs on dendrites of CA1 hippocampal pyramidal cells (226–228). There is also evidence to suggest that synaptic pruning is influenced by neurosteroids during puberty. Optimal spine density may depend in part on expression and modulation of α4βδ GABA_A_Rs during puberty, which in turn appears necessary for optimal learning and memory function in adulthood (229). The estrus cycle influences memory function in female rodents (230). Spatial learning and memory deficits observed on the morning of proestrus phase in rodent are associated with an increased expression of α4βδ GABA_A_Rs on CA1 pyramidal cell dendrites (230). There is a reversible decrease in the expression of δ-GABA_A_Rs in parvalbumin containing interneurons in the CA3 hippocampal subregion during pregnancy in rodents, and this change is associated with increased levels of ALLO (231). Reduced expression of δ-GABA_A_Rs and increased ALLO may serve to counterbalance each other during pregnancy so that tonic inhibition is maintained. It has been suggested that dysregulation of this delicate balance alters cortical activity in the γ frequency range and that measuring drug-induced changes in cortical γ activity could serve as a non-invasive objective biomarker for predicting the efficacy of pharmacological interventions (231). A non-invasive objective biomarker of this type could prove to be very useful in open label studies.

### Timing of Neurosteroid Administration in Relationship to Effect on Memory Function

Learning and memory function can be broken down into three phases: (1) acquisition; (encoding); (2) consolidation; and (3) retrieval (recall). As a result, in addition to their unique and sometimes complex mechanisms of action, the timing of neurosteroid administration can also influence performance on specific tests sensitive to the different aspects of learning and memory function. For example, post-acquisition administration of PregS is associated with improved retention in rodents on a passive avoidance paradigm, indicating that this positive modulator of glutamatergic neurotransmission facilitates consolidation (206). Systemic administration of PregS enhances acquisition (31), while direct infusion of this neurosteroid into the lateral septum 30 min before training appears to interfere with acquisition (185). This seeming discrepancy between the two studies can likely be explained by the complexity of the septo-hippocampal interaction and Pregs-induced increase in excitation and/or decreased excitation within the septum (232).

### Specific Type of Memory Function Being Assessed

But what about the specific type of memory function being assessed with a specific task; how might this affect interpretation of behavioral response to neurosteroids? Different types of memories require activation of different brain regions. Regional changes in activation are also associated with specific neuropsychiatric and neurologic disorders. Problems with attention, concentration, and motivation can interfere with encoding of new information and, thus, with performance on tests of memory function (233). While the hippocampus is involved in most aspects of learning and memory function, certain types of memory appear to depend less on activation of this brain region than on other regions (234).

The role of neurosteroids in semantic memory function can only be effectively studied in humans who have the inherent ability to verbally express what they know about the world (235). Humans are also capable of using non-episodic strategies on tests looking at the temporal aspects of an episodic memory (236). This distinction can be very important when parsing out the effects of neurosteroid on episodic vs. semantic memory function. For example, systemic administration of ALLO impairs episodic memory but not working or semantic memory function in healthy adult women (203). The effects of neurosteroids on semantic memory function in schizophrenia has not been elucidated; however, PREG is metabolized to ALLO and PregS, both of which appear to improve working memory function in this patient population (51, 52, 65, 237, 238). Whether this effect on performance is mediated by PREG, ALLO, or PregS alone or via a combination of all three neurosteroids has not been determined.

The effects of neurosteroids on learning and memory for fear-inducing stimuli appears to be different in males and females, who show different brain levels of ALLO at baseline as well. Augmenting levels of ALLO in the bed nucleus of the stria terminalis (BNST) of males, who have lower levels of ALLO at baseline, promotes contextual freezing. By contrast, reducing intra-BNST levels of ALLO in females, who have higher brain ALLO levels at baseline, enhances the expression of contextual freezing (239). In addition to differences in responding due to sex-related baseline levels of neurosteroids, different aspects of memory function depend on recruitment of different brain regions, as well as on activation of different subregions within the hippocampus (e.g., dorsal vs. ventral hippocampus) (240).

Learning and memory of coordinated motor activities involves activation of the cerebellum where neurosteroids are synthesized *de novo* by Purkinje cells (241–243). It has recently been suggested that 17β-estradiol may also play a role in cerebellar motor memory formation in male rats (244). Animal studies also suggest that the glutamate-nitric oxide-cyclic GMP pathway is impaired in the cerebellum and cortex of subjects with hyperammonemia due to hepatic failure. Ammonia-induced disruption of this pathway has been implicated in impaired performance on certain types of procedural tasks that depend in part on optimization of motor performance. Restoring the pathway and cyclic GMP levels in the brain restores learning ability. The role of neurosteroids in cerebellar function and the acquisition of motor skills has not been fully elucidated. Elevated levels of GABAergic neurosteroids have been associated with hepatic failure, and it has been suggested that PregS may be of clinical benefit in the treatment of deficits in motor coordination and memory disturbances associated with hyperammonia seen in this patient population (245).

Where within the pharmacological connectome do neurosteroids act to modulate different types of learning and memory function *in vivo*? Not too surprisingly, direct infusion of agonists and antagonists into the brain regions implicated in these different types of memory can either augment or inhibit learning and memory function. For example, the effects of the neurosteroid ALLO on acquisition and extinction of memories associated with exposure to powerful emotion-evoking or fear-inducing stimuli are state independent when it is injected into the amygdala and hippocampus, but state-dependent when injected into the BNST (246). Due to the high concentration of GABA_A_ receptors in the basolateral amygdala, infusion of GABAergic neurosteroids, such as ALLO into this brain region interferes with the acquisition and expression of the contextual and auditory cue-induced freezing responses in male rats (246).

### Age and Cognitive Status of Subjects at Time of Testing

Neurosteroids are implicated in age- and disease state-dependent impairments in learning and memory function (247–249). Because aging subjects can be comorbid for neuropsychiatric disorders, such as depression and age-related neurodegenerative diseases that both affect memory function (250), understanding the role of neurosteroids in age-related changes in memory function can shed light on potential therapeutic targets suitable for this unique patient population.

Low brain levels of ALLO have been associated with memory impairments in aged rats as well as in human subjects diagnosed with AD (67, 248). In addition, post-mortem studies of human subjects with AD reveal increased brain levels of DHEA and PREG (67). Autopsy studies in humans indicate that brain testosterone levels are lower in patients with AD in comparison to normal men (251) and free testosterone has been implicated as a risk factor for probable AD based on clinical diagnostic criteria (252). However, although low testosterone has been implicated in cognitive deficits in healthy men, testosterone replacement therapy does not appear to significantly improve memory function in cognitively impaired older men with low testosterone (253). It is important to point out that although *in vivo* biomarkers are expected to enhance the pathophysiological specificity of the diagnosis of AD dementia in future studies, most published studies to date looking at the role of neurosteroids on memory function have used core clinical criteria for the diagnosis of possible/probable AD type dementia. Interpretation of such results is hampered in human studies as inclusion of subjects who will ultimately not meet the definitive diagnostic criteria for AD at autopsy is a possible confounding factor.

Decreased plasma levels of DHEA and DHEAS have also been reported in humans with AD (71). It has been suggested that patients meeting clinical criteria for probable AD who have higher baseline levels of DHEAS may perform better on memory tasks than those with lower levels, while by contrast patients with lower circulating levels of cortisol may perform better than those with higher plasma cortisol (68). Men with probable AD based on clinical features of the disease show diurnal changes in cortisol levels characterized by a significant increases at 03 h 00 not seen in healthy elderly men. This increase in cortisol levels occurs despite a slight decrease in levels of adrenocorticotropic hormone (70). Increased levels of DHEA, adrenocorticotropic hormone (ACTH), and interleukin-6 (IL-6) are seen in the morning in women diagnosed with clinical symptoms consistent with AD (70). Cortisol levels are important to the different sleep stages.

Although sleep disturbances are implicated in AD, it is not entirely clear how the aforementioned diurnal changes in cortisol influence sleep and memory consolidation. Sleep plays a role in memory consolidation, and neurosteroids, such as PregS and ALLO can modulate activity within brain regions, such as the pedunculopontine tegmentum nucleus implicated in regulation of sleep (248, 254, 255). Neurosteroids, such as PregS have been implicated in disturbances of sleep and cognitive dysfunction in AD rodent models (247, 256). It has been suggested that the promnestic effects of PregS are mediated by an increase in paradoxical (a.k.a., rapid eye movement; REM) sleep (257, 258), but whether or not the effects of PregS on memory function in subjects with neuropsychiatric disorders and age-related neurodegenerative disease are mediated by enhancement of REM and/or non-REM sleep has not been fully elucidated (259, 260).

Positron emission tomography (PET) imaging studies using the high-affinity sigma-1 (σ-1) receptor selective PET tracer [18F]1-(3-Fluoropropyl)-4-[4-cyanophenoxy) methyl]piperidine ([18F]FPS) as a radioligand suggest that neurosteroids including DHEA bind to σ-1 receptors *in vivo* (261). Sigma-1 receptors modulate NMDA-mediated responses (262, 263). Agonists of the σ-1 receptor, which acts as a molecular chaperone on mitochondria-associated endoplasmic reticulum membranes, appear to provide neuroprotection in rodent models of AD (264).

PET scans using the radiotracer [carbonyl-(11)C]WAY-100635 indicate that progesterone and DHEAS modulate serotonin 1A (5-HT1A) receptor binding *in vivo* (265, 266). While there is evidence of disrupted serotonergic neurotransmission in AD (267), the role of neurosteroids, such as DHEAS in the progression of this age-related neurodegenerative disease has not been fully elucidated.

Hyperactivity of entorhinal cortical and hippocampal circuits are also thought to underlie neurodegenerative disorders, such as AD and mild cognitive impairment (MCI) (268–272). Early synaptic dysfunction or “synaptopathy” at the level of inhibitory interneurons within the entorhinal cortex and CA3 hippocampal subregion leads to hyperactivity of pyramidal cells, which appears to play a role in the progression of AD neuropathology (e.g., tauopathy) (271, 272). This hyperactivity may be due in part to reduced responsiveness of pyramidal neurons to GABAergic inhibitory inputs (272). Other studies implicate a loss of GABAergic interneurons in aging rat models of MCI, suggesting that multiple mechanisms may play a role and/or may interact to contribute to hyperactivity (273, 274). Studies using human tissue from patients with late stage AD reveal hippocampal subregion- and strata specific changes in receptor subunit expression that could potentially influence memory function (275). For example, α5 subunit expression, which is implicated in learning and memory function, is increased in the pyramidal layer and oriens of the CA1 subregion. In addition, expression of the α1 subunit, which is implicated in sedation, is increased in all strata of the CA3 subregion and in the granule cell layer and hilus of the dentate gyrus. An increase in α2 subunit expression, which has been implicated in anxiety, is seen in the oriens and radiatum in the CA3 subregion. Expression of α2 is increased in the oriens of the CA1 subregion, but is decreased in the pyramidal layer. There is a decrease in β3 subunit expression in the granular and molecular layers of the dentate gyrus, while expression of α3 and β1 subunits remain unchanged.

ALLO may be useful in attenuating the hyperactivity implicated in early stage AD (201, 276). In mouse models, ALLO appears to increase neurogenesis, reduce amyloid deposition and improve performance on learning and memory tests, suggesting that it may serve as a regenerative therapeutic (197, 277–279). A clinical trial for ALLO is ongoing. Other investigations indicate that chronic treatment with riluzole, which includes a reduction in glutamatergic neurotransmission among its mechanisms of action, attenuates the spread of tauopathy in rodent models (280). Is it possible that neurosteroids, such as pregnanolone sulfate and its synthetic analog pregnanolone hemisuccinate, which negatively modulate glutamatergic neurotransmission, may also have potential therapeutic applications in AD and age-related MCI.

Upregulation of TSPO expression has been implicated in neuropsychiatric disorders and neurodegenerative disease (281–283). Oxidative stress is associated with induction of neurosteroid biosynthesis in the human brain (284), and TSPO ligands (*N*,*N*-dialkyl-2-phenylindol-3-ylglyoxylamides) that also show anxiolytic activity promote a reduction in oxidative stress and pro-inflammatory enzymes in glial cells via promotion of neurosteroid synthesis (133). The role of this pathway in the progression of neurodegenerative diseases associated with memory impairments, such as AD has not been fully elucidated. However, increasing oxidative stress by treating oligodendrocytes with beta-amyloid is associated with an increase in the synthesis of DHEA (284), which provides some neuroprotection in a rodent model of AD (285).

Radioligands are being used to measure TSPO expression *in vivo*. PET scans using 11C-PBR28 as a radiolabeled tracer indicate that binding to TSPO is greater in patients with early-onset AD than in those with late-onset disease. Binding to TSPO is also inversely correlated with gray matter volume and performance on measures of cognitive function in all patients with AD. Early-onset patients have greater 11C-PBR28 binding than late-onset patients. Additionally, an increase in TSPO binding is not seen in patients with age-related MCI, even though these patients show increased amyloid pathology as assessed by Pittsburgh Compound B PET scans and hippocampal atrophy as assessed by volumetric analysis of MRI scans (282). The largest differences in TSPO binding in these groups are seen in the temporal and parietal cortices.

These findings collectively point to a potential therapeutic use for neurosteroids in age-related neurodegenerative diseases. The unique modulatory properties of neurosteroids make these particularly well-suited for targeting comorbid anxiety and depression as well as memory deficits in these patient populations.

## The Role of Neurosteroids in Memory Deficits Associated With Stress and Anxiety Disorders

Three large scale major neural networks—the default mode network, central executive network, and salience network—contribute to cognitive processing within the human brain and function together to facilitate adaptive responses of the CNS (286). The *default mode network* plays a role in episodic memory function and self-related cognitive activities including autobiographical memories. Key functional nodes within the *default mode network*, which includes the hippocampus, amygdala, and medial prefrontal cortex, have been implicated in AD and epilepsy (287, 288). The frontal parietal connections of the *central executive network* control attention, working memory, and executive function.

Connections within the central executive network have been implicated in schizophrenia (289). Responses to emotional changes and reward stimuli are dependent on intact function of the *salience network*, within which the anterior insula, anterior cingulate cortex and amygdala, ventral tegmental area, and thalamus function together to segregate the most relevant and rewarding among internal and extra-personal stimuli in order to guide behavior. Stronger connectivity within the salience network has been associated with increased anxiety (290, 291). Pathological activation of the salience and default networks can interfere with the process of switching between these two networks, which would have a differential impact on mood and memory functionality. Neurosteroids can therefore differentially modulate these three major neural networks central to memory processing.

**Figure 11:**
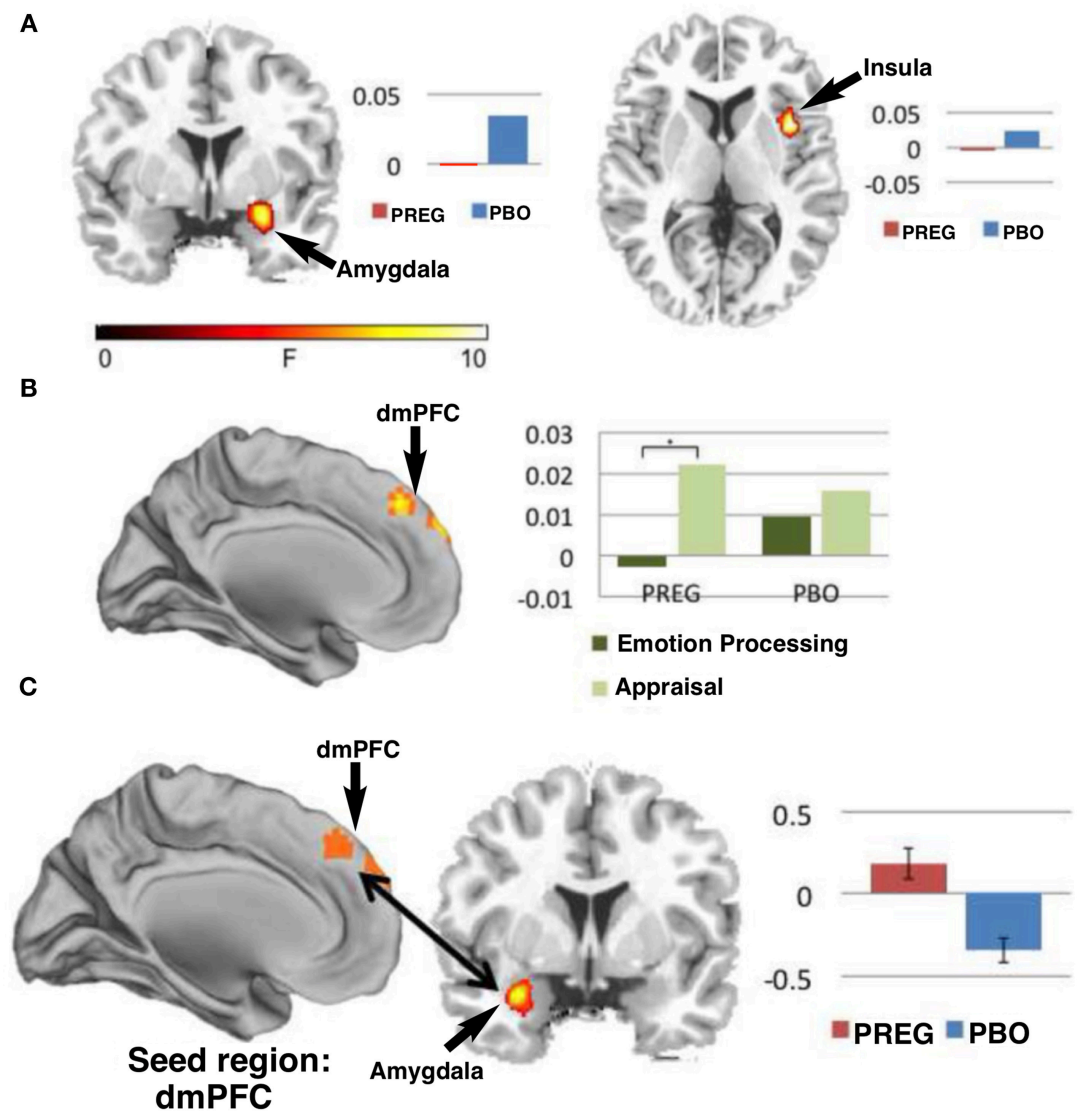
Functional MRI studies show that treatment with PREG enhances activation of neural circuitry involved in emotional regulation as measured by a Shifted-Attention Emotion Appraisal (SEAT) paradigm. The SEAT task is designed to probe multiple aspects of emotional regulation in response to presentation of images depicting neutral, angry, or fearful expressions superimposed on pictures of indoor or outdoor scenes. Sixteen subjects were treated with PREG and 15 were treated with placebo (PBO). Subjects viewed the stimuli through MR-compatible goggles and responded using an MRI-compatible button box. Maps of cerebral activation in each condition indicated that: **(A)** There was a significant (*p* < 0.05) main effect of drug in the amygdala [Montreal Institute (MNI) coronal (y) plane coordinate = 2] and right insula [MNI axial (z) plane coordinate = −6] such that, treatment with PREG decreased activity in these two regions across all conditions and face types. **(B)** Treatment with PREG increased activation in dorsal medial prefrontal cortex (dmPFC) [MNI sagittal (x) plane coordinate = 0] during appraisal. **(C)** PREG treatment also significantly (*p* < 0.001) increased functional connectivity between the dmPFC and left amygdala during appraisal. Self-reported anxiety was inversely correlated (*r* = −0.52, *p* = 0.046) with functional connectivity between the dmPFC and amygdala in the PREG group. Percent signal change is displayed next to each figure [Modified from Sripada et al. (192) with Permission].

**Figure 12:**
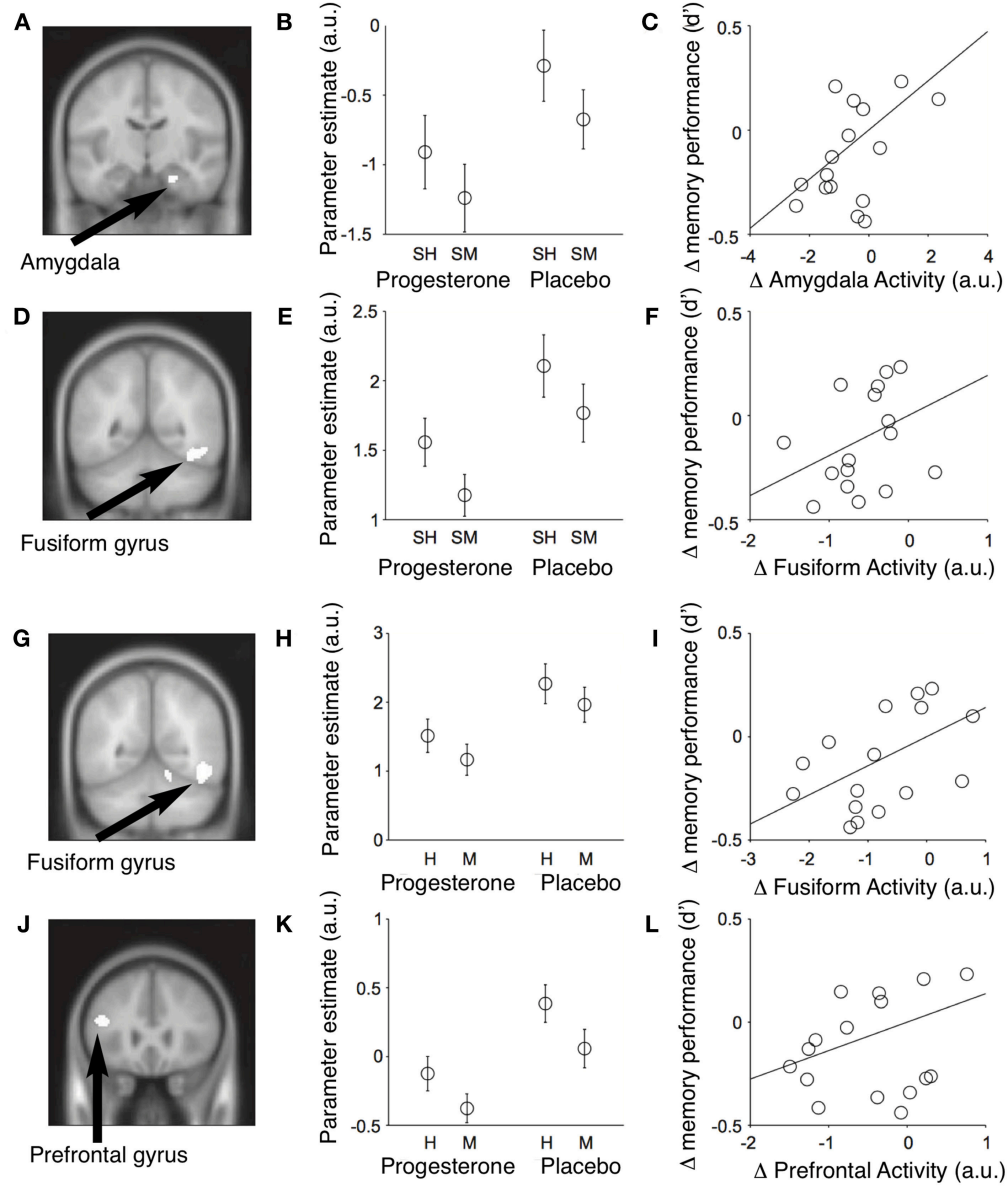
Event-related fMRI studies in female subjects showing effects of progesterone on brain regions involved in the encoding and retrieval of memories of faces. Results of conjunction analyses for the relationships between neural activity in different brain regions and memory performance across subjects. **(A)** During encoding of memories for faces, progesterone administration was associated with reduced activity in the right amygdala (MNI coronal (y) plane coordinate = −12; *p* < 0.001, uncorrected) and fusiform gyrus [MNI coronal (y) plane coordinate = −52] which predicted a decrease in memory performance across subjects. **(B)** Parameter estimates for mean activity within each significant conjunction cluster (SH = subsequent hits; SM = subsequent misses; a.u. = arbitrary units; mean ± SEM). **(C)** The progesterone-induced decrease in amygdalar activity predicted the decrease in memory performance across subjects [recognition memory accuracy = d′]. **(D–F)** Identical figures for the significant conjunction of main effects in the right fusiform gyrus and memory performance. Progesterone reduces activity in the fusiform and inferior frontal gyri and impairs performance during retrieval of memories for faces**. (G)** Significant conjunction of main effect of memory (i.e., hits > misses) and main effect of drug [i.e., placebo (faces > null events) > progesterone (faces > null events)] in the right fusiform gyrus [MNI coronal (y) plane coordinate = −56; *p* < 0.001, uncorrected]. **(H)** Parameter estimates of the conjunction cluster (arbitrary units; mean ± SEM). **(I)** Progesterone-induced reduction in fusiform gyrus activity predicted the decrease in memory performance across subjects [recognition memory accuracy (d′)]. **(J–L)** Identical figures for the significant conjunction of main effects in the left inferior frontal gyrus [MNI coronal (y) plane coordinate = 26]. H, Hits; M, misses; a.u., arbitrary units. H, Hits; M, misses; a.u., arbitrary units [Modified from van Wingen et al. (64) with Permission].

Neurosteroids play a role in anxiety and in the learning and memory deficits associated with certain anxiety disorders. Treatment of subjects under acute psychosocial stress with DHEA (50 mg/day) both improves attention but also impairs declarative memory function (78), suggesting that the benefits in one cognitive domain may be offset by deficits in another at this dose. Neurosteroid modulation of GABAergic neurotransmission in the central amygdala has been implicated in anxiety (131) and the effects of ALLO on anxiety appear to be mediated in part via modulation of activity within the amygdala, which in turn influences neural activity in brain regions involved in learning and memory function (64, 192, 292) (Figures 11, 12). Although the role of 5α-reductase inhibition in the memory deficits associated with anxiety disorders has not been fully elucidated, it has nevertheless been suggested that serum and brain levels of ALLO are increased by 5α-reductase inhibition.

The BNST, which is also referred to as the extended amygdala, receives input from the hippocampus and shares reciprocal projections with the paraventricular nucleus of the thalamus (246, 293–296). This brain regions play a role in adaptive responses of the hypothalamic-pituitary-adrenal axis (HPA axis) to fear-inducing stimuli (239, 246, 296). The effects of ALLO on acquisition and extinction of hippocampal-dependent memories associated with fear-inducing stimuli appear to be state-dependent following direct injections of this neurosteroid into the BNST. Direct injections of ALLO into the BNST during conditioning or testing suppressed contextual fear, but this effect was not seen when the neurosteroid was injected into the BNST during both procedures (246).

Selective serotonin reuptake inhibitors (SSRIs) increase brain levels of ALLO in humans and animals, suggesting that the anxiolytic effects of SSRIs may be related in part to their effect on CNS levels of this endogenous GABAergic modulator (93, 297–301). Patients with anxiety disorders often self-medicate with ethanol. Acute exposure to ethanol also increases brain levels of ALLO (302). By contrast, preclinical studies in a rat model suggest that chronic intermittent exposure to ethanol is associated with decreased ALLO levels in the hippocampus. Although protein levels were not reported, in this study, mRNA levels for 5alpha-reductase and 3alpha-HSD were noted to be reduced in the hippocampus of these rats, which also had impaired performance on a hippocampal-dependent memory test and increased sensitivity to the anxiolytic effects of alphaxalone (303). Could targeting neurosteroidogenesis (304) and/or steroid metabolism be a viable strategy for the treatment of patients presenting with comorbid anxiety and alcohol use disorders?

When compared with normal subjects and patients with major depression, elderly patients with generalized anxiety disorder (GAD) show more deficits on tests of short-term memory function (305). The severity of GAD has been found to positively correlate with cortisol levels in saliva of older adults (73). Elevated salivary cortisol in older adults is associated with impaired performance on tests of memory function (306). Treatment of older adults presenting with GAD and elevated baseline cortisol with SSRIs is associated with a reduction in salivary cortisol that correlates with reductions in anxiety (307). SSRIs increase levels of ALLO (299). and ALLO restores hippocampal-dependent learning and memory function in a rodent model of AD (308). AD is associated with non-cognitive behavioral and psychological symptoms, which can include paranoia and anxiety; therefore, ALLO may be well-suited for targeting these as well as the cognitive deficits associated with this form of dementia (309).

Cortisol seems to enhance treatment outcomes in a small group (males and females) treated with exposure-based group therapy (310). In addition, high endogenous estradiol vs. low estradiol correlates with better treatment outcomes in exposure therapy (311) in female patients with spider phobias. Investigations of neural network activity using intracranial electroencephalography in human subjects indicate that β-frequency coherence (13–30 Hz) between the amygdala and hippocampus encodes variations in mood (312). Stress may mediate changes in mood and cognition in early adolescence and may play a role in the expression of psychopathologies in adulthood. During stress, corticotropin-releasing hormone promotes an increase in adrenocorticotropic hormone release. This promotes an increase in the concentration of cholesterol at the inner mitochondria membrane of the adrenal cortical cell via activation of the steroidogenic acute regulatory protein. Cholesterol is then converted to PREG and its steroid metabolites, including PregS, cortisol, corticosterone, DHEA, and ALLO (77, 87, 313, 314).

DHEA and DHEAS appear to counteract the negative effects of increased cortisol on working memory function in women and men, respectively (315, 316). A dose-dependent inverted U-shaped response to DHEAS is observed on tests of learning and memory function in male mice (30). By contrast, DHEA is effective in a wider range of doses, suggesting it would be a better choice as a therapeutic (30). Although a positive correlation between DHEAS and cognitive function has been observed in women and men, more research is needed to determine if the effects of DHEA and DHEAS on learning and memory function in humans are sex, dose, and disease state dependent (30, 317, 318).

The response to stress appears to be influenced in part by circulating steroid hormones. Stress increases associative learning and dendritic spine density in the hippocampus of male rats, but impairs associative learning and reduces spine density in females (319). The potential effects of neurosteroids on development of anxiety disorders can be observed during puberty, which is not only associated with increase in levels of reproductive hormones but also with the onset of many psychiatric disorders, including GAD, social anxiety, and panic attacks (320–326). Anxiety symptoms have been found to increase from middle to late adolescence (323), with a particularly high prevalence of all anxiety disorders reported among adolescent girls (324, 327, 328). Of particular interest in this setting are the neurosteroids ALLO (in human and rat) and pregnanolone (in human only), metabolites of the reproductive hormone progesterone that are also produced in the brain in response to stress (79, 329).

Animal studies suggest that developmental exposure to ALLO influences subsequent responsivity to anxiolytics in adulthood (206, 330–332). Acute stress is associated with an increase in plasma ALLO levels that correlates with an increase in expression of the TSPO, also known as the peripheral benzodiazepine receptor. Because the TSPO plays a role in steroidogenesis, it has been considered as a therapeutic target for treatment of anxiety disorders (77, 333). It is interesting to note, that ALLO levels have been found to be decreased relative to controls in the CSF of women with chronic stress disorders, such as PTSD (75). A subsequent study from this group observed a negative correlation between CSF levels of total GABAergic neurosteroids levels (ALLO plus pregnanolone) and PTSD symptoms in men (76). This study also found an association between PTSD and reduced 5α-reductase mediated biosynthesis of ALLO in men, which is in contrast to the block at 3α-HSD previously observed in women with PTSD. Other studies have found that administration of sodium lactate and cholecystokinin tetrapeptide to persons diagnosed with panic disorder decreases plasma concentrations of both pregnanolone and ALLO, and increases the concentration of the functional antagonistic isomer 3β,5α-tetrahydroprogesterone (334). These findings suggest that both acute stress and chronic stress give rise to unique effects on ALLO levels in men and women. Although studies in post-menopausal women did not reveal any benefit on tests of short-term memory function, it has nevertheless been suggested that replenishing this neurosteroid may have beneficial effects on memory function in certain populations with age-related memory deficits (335, 336).

Restoration of altered endogenous neurosteroid levels via modulation of steroidogenesis mediated by pregnane xenobiotic receptors (PXR) and the endocannabinoid system has been suggested as an alternative to direct administration of neurosteroids (89, 178, 337). Because PREG is a precursor for all steroid hormones, it seems plausible that promotion of cortisol synthesis during stress may attenuate synthesis of other steroid hormones, but this suggestion has not been substantiated.

These findings suggest that changes in endogenous brain levels of neurosteroids associated with age, sex, stress, and administration of SSRIs may play a key role in the onset and clinical response to pharmacologic interventions in certain anxiety disorders. Supporting this hypothesis are findings related to the effects of ALLO on gonadotrophin-releasing hormone (GnRH), the primary chemical messenger implicated in the onset of puberty and sexual maturation (338). Central precocious puberty is associated with ALLO, which has been found to suppress the release of hypothalamic GnRH via allosteric modulation of GABA_A_Rs (339, 340). Although inhibition of GnRH release is increased by ALLO administration before puberty and in adulthood, it is paradoxically reduced during puberty (212).

The reduction in GnRH release during puberty is also associated with increased excitability of pyramidal cells in hippocampal region CA1. This effect appears to be due in part to inhibition of α4-containing GABA_A_Rs, which are expressed at higher levels than normal in the CA1 region of the hippocampus during puberty. GABA_A_Rs of the α4β2δ subtype, which have a δ subunit instead of a γ subunit, play a role in tonic inhibition in areas, such as the dentate gyrus and cortex (341). GABA_A_R-mediated conductance is normally inhibitory; however, the reversal potential of GABA_A_R-mediated post-synaptic current in dentate gyrus granule cells is “positive” to the resting membrane potential, making membrane hyperpolarization of GABA_A_Rs unlikely in this region. Inhibition of shunting appears to play a role in overcoming this process, such that non-hyperpolarizing inhibitory conductance reduces the depolarizing effect of post-synaptic potentials by decreasing proximal membrane resistance (342). In the dentate gyrus and cortex, the GABAergic current is inward (i.e., chloride flux is outward) (342, 343), and thus inhibition in these areas is enhanced by ALLO. However, in the CA1 hippocampal subregion the current is normally outward (344), and thus increased expression of α4β2δ GABA receptors paradoxically results in ALLO attenuating rather than enhancing inhibition. The reduction in currents generated by ALLO at α4β2δ GABA_A_R is dependent upon the presence of arginine 353 in the intracellular loop of α4, where it may serve as a chloride modulatory site (345). This polarity-dependent decrease in inhibition mediated by ALLO may have important implications for how we approach the memory deficits associated with anxiety disorders, which may prove to be amenable to therapeutic strategies targeting the expression and/or activity of GABA_A_Rs of the α4β2δ subtype (346).

## The Role of Neurosteroids in Memory Deficits Associated With Depression

Changes in neurosteroid levels have been implicated in onset of depression and in the actions of medications used to treat depression. Animal studies using a synthetic analog of ALLO suggest that fluctuations in neurosteroids levels may also influence motivation to learn via modulation of dopaminergic pathways (347). The effects of ALLO on mood in women appear to follow an inverted U-shaped curve (214). SSRI treatment has been associated with increased brain levels of ALLO, suggesting that the memory enhancing effects of SSRIs in this patient population may be related in part to the actions of this neurosteroid (93, 297, 348, 349). Preclinical animal studies suggest that modulation of GABAergic neurotransmission by DHEA may also have therapeutic potential in the treatment of memory deficits associated with depression (350).

It is not entirely clear how ALLO, which is a positive allosteric modulator of GABAergic neurotransmission, acts to improve memory function in this patient population, but it has been suggested that stress-induced changes in GABA receptor expression levels are likely to play a role in the etiology of depression. This hypothesis is supported by preclinical studies showing that early life traumatic stress is associated with chronic anxiety, spatial memory deficits and reduced expression of GABA_A_R subunits in the adult rat brains (351).

It has been suggested that PREG and its metabolites may be efficacious in the treatment of depressive disorders. Treatment of depression poses several challenges and this is especially true in the treatment of depression in bipolar disorder (BPD). In a study conducted by Brown et al. (352), 80 adults with BPD and depressive mood state were treated with PREG or placebo as add-on therapy for 12 weeks. Outcome measures included the 17-item Hamilton Scale for Depression, Hamilton Rating Scale for Anxiety (HRSA) and Young Mania Rating Scale. Assessment of serum neurosteroid levels at baseline and treatment completion (week 12) revealed large baseline-to exit changes in neurosteroids in the PREG treatment group. In the PREG group, unlike the placebo group, HRSA changes negatively correlated with ALLO and PREG levels, indicative of reduced anxiety. The results of this small study should be interpreted with caution because subjects were taking a wide variety of medications including lithium, antidepressants (unspecified), sedative hypnotics/anxiolytics (unspecified), antipsychotics (unspecified), and stimulants (unspecified) and they were not stratified based on the drugs they were taking for their depression.

## The Role of Neurosteroids in Memory Deficits Associated With Schizophrenia

The cognitive deficits associated with schizophrenia are caused by multiple factors, and elucidating a single causative part of the cognitive component would provide some hope for understanding memory and a basis for therapeutic discovery. Neural circuitry-based studies highlight cortical disinhibition as a critical factor in schizophrenia affecting GABAergic interneurons (parvalbumin, SOM/NPY/CCK/expressing interneurons). GAD67 deficiencies together with changes in GABA_A_R expression, in particular the α2 subunit, are known (353–356). Hypofunction of NMDARs, particularly in inhibitory parvalbumin (PV) interneurons (357), may initiate the disease process, leading to decreased inhibition of pyramidal neurons. NMDAR hypofunction in inhibitory interneurons is implicated in the observed GABAergic deficits (358–364). Whatever the specific path for initiating dynamical imbalance or dysregulation of the circuitry, the resultant hyperactivity of downstream pyramidal neurons ultimately leads to dysregulated neural network activity, excitotoxicity, and eventually frank neuronal loss. Note that hypofunction of NMDARs expressed in pyramidal neurons themselves is also likely (365). Disruptions of neural network activity during sleep, including reduced sleep spindle activity, has been associated with impaired sleep dependent memory consolidation in schizophrenia (260).

Synchronous activity of PV interneurons generates gamma oscillations which are observed during performance of cognitive tasks. Cognitive deficits are thought to arise from a disturbance of these high frequency oscillations in the gamma range due to reduced excitatory drive to cortical PV interneurons (366, 367) with resultant network hypersynchrony (368, 369). Manipulations of various receptors such as α5 type GABA_A_Rs and sodium channels have been shown to enhance cognition (356). The glutamate hypothesis of schizophrenia underscores the importance of NMDARs in neuropsychiatric disorders. NMDARs are critical for the generation of gamma oscillations, and dysfunctional NMDARs result in psychosis and deficits in specific cognitive domains. Genetically engineered mice lacking NMDARs show deficits in habituation, working memory and associative learning (370).

Enhancing NMDAR function is therefore vital for enhancing cognition in healthy individuals and those with neuropsychiatric disorders (111). However, attempts to enhance NMDAR neurotransmission thus far have not been successful (371). A recent meta-analysis of currently available NMDAR positive allosteric modulators reveals that these are ineffective in alleviating cognitive impairments (371). Maintaining a critical balance in NMDAR activation is optimal for avoiding the negative consequences of NMDAR stimulation a, making PregS, DHEAS and their analogs highly suitable candidates. Subunit selective modulation, combined with its ability to potentiate receptor function without overstimulation, renders PregS a likely and highly suitable positive modulator of NMDARs (111, 371).

PREG and ALLO have been investigated as potential therapeutics for treating memory deficits associated with schizophrenia (50, 65, 238). Acute administration of either clozapine or olanzapine increases brain and plasma levels of PREG, which is a precursor of ALLO and PregS (372). Adrenalectomy prevents clozapine-induced increase in hippocampal PREG. By contrast, significant increases in PREG levels in rat hippocampus are not observed following acute administration of aripiprazole, quetiapine, or ziprasidone administration. This suggests that the effects of second-generation antipsychotics on memory function may be due in part to increased brain levels of PREG and/or PregS (28, 48, 51, 62, 373).

We have previously demonstrated that low picomolar concentrations of PregS are sufficient to increase [Ca^2+^]i and CREB phosphorylation (48). There is some evidence that sulfotransferase 4A1 haplotype 1 (SULT4A1-1)-positive subjects show a better response to olanzapine than do SULT4A1-1 negative subjects. However, the exact role this sulfotransferase isozyme plays in the formation of PregS from PREG and the amelioration of learning and memory deficits in humans with schizophrenia treated with second-generation antipsychotics has not yet been established (374). ALLO-mediated modulation of GABAergic neurotransmission has also been implicated in the antipsychotic effects of clozapine and olanzapine in rodent models (375–377).

Multiple clinical and preclinical studies have examined the effects of neurosteroids, PREG, PregS and DHEA on memory and other cognitive attributes. Cognitive deficits are a core feature of schizophrenia (378). Recent clinical trials in patients with schizophrenia suggest that treatment with PREG as add-on therapy alleviates cognitive deficits (50). In patients receiving PREG, plasma levels of its immediate metabolite, PregS (positive modulator of NMDARs) and ALLO (positive modulator of GABA_A_Rs) are elevated, suggesting a possible role for PregS in improvement of learning and memory function (50). Earlier studies (28) had also described the cognition enhancing effects of PregS (29).

### Neurosteroids, Dopamine Knockout Mouse Model and Schizophrenia

Dopamine (DA) is a neuromodulator and a key player in several neural disorders in which cognitive deficits are characteristic, such as schizophrenia, attention deficit hyperactivity disorder, and depression (379–381). The dopamine transporter (DAT) mediates re-uptake of extracellular DA, thus terminating DA receptor activation. DAT, a member of the Na^+^/Cl^−^-dependent family of neurotransmitter transporters (382) is therefore a major therapeutic target for schizophrenia and other disorders (383–385). Several transgenic models have been generated with changes in DAT expression or function or mutations in the DAT gene (386). These models have proven to be crucial in elucidating neurotransmitter and neuromodulator function (or mechanistic underpinnings) of neuropsychiatric disorders (381, 387).

Wong et al. (373) have used a DAT knockout (DAT KO) mouse model exhibiting symptoms characteristic of schizophrenia to investigate the effects of PregS treatment on learning and memory function. Systemic administration of PregS, which is able to cross the BBB, alleviates positive and negative symptoms as well as cognitive deficits in the DAT KO mouse. Long-term systemic treatment with PregS rescues impaired episodic memory and poor discriminative abilities in the DAT KO mice without adverse effects. Consistent with observed reductions in cognitive deficits, long-term PregS treatment increases expression of the obligatory NMDAR subunit GluN1 in the hippocampus. Earlier, our laboratory characterized some of the mechanisms involved in increased expression of NMDAR subunits and demonstrated that PregS increases surface GluN1 in oocytes expressing recombinant NMDAR subunits in a non-canonical GPCR- and Ca^2+^-dependent manner (47) (Figure 2).

## Synthetic Neurosteroids as Potential Cognitive Enhancers

**Figure 13:**
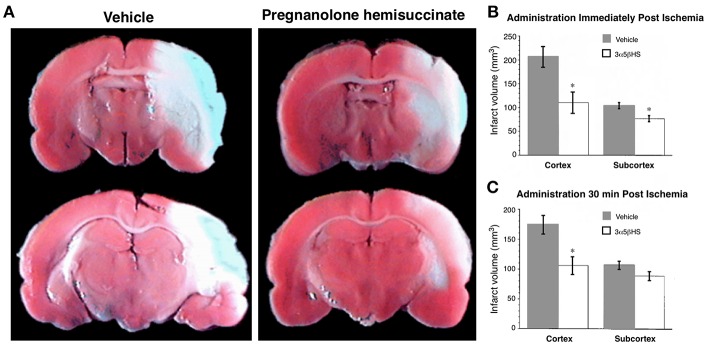
Pregnanolone hemisuccinate (3α5βHS) is neuroprotective in an *in vivo* model of stroke. Rats were infused with either vehicle or 3α5βHS (6.9 mg/kg/h; 6.9 mg/kg loading dose), beginning immediately or 30 min after initiation of medial cerebral artery occlusion. Infusion of 3α5βHS was continued for an additional 22 h, at which time the rats were euthanized and their brains harvested and stained with 2,3,5-triphenyl tetrazolium Cl. **(A)** Representative coronal sections from animals receiving vehicle or 3α5βHS infusions immediately after initiation of ischemia. Infarct area appears pale. **(B)** Administration of 3α5βHS immediately after the onset of ischemia significantly reduced the volume of the cortical infarct from 206 ± 22 to 110 ± 21 mm^3^ (*P* < 0.005) and subcortical infarct was reduced from 103 ± 6 to 76 ± 6 mm^3^ (^*^P < 0.005) (vehicle, *n* = 9; 3α5βHS, *n* = 10). **(C)** When 3α5βHS was administered 30 min after the onset of ischemia the volume of cortical infarct was reduced from 173 ± 15 to 106 ± 15 mm^3^ (^*^P < 0.005; *n* = 13), with no reduction apparent in the subcortical region [From Weaver et al. (132) with Permission].

The findings reviewed herein suggest that the memory deficits seen in patients with schizophrenia, depression and anxiety disorders are influenced by changes in endogenous neurosteroid levels. Because not all systemically administered endogenously occurring neurosteroids readily cross the BBB and because these compounds can be metabolized to hormonally active steroids with different mechanisms of action, the therapeutic potential of these compounds is limited. For this reason, synthetic analogs of neurosteroids, which are more resistant to metabolism and better able to cross the BBB, are under investigation for use as anxiolytics, antidepressants, cognitive enhancers, anesthetics, and anticonvulsants (132, 388, 389). For example, synthetic neuroactive steroids bearing a hemisuccinate group are more resistant to hydrolysis than the corresponding sulfate esters and are partly unionized at physiological pH, allowing increased passage across the BBB. One such synthetic neuroactive steroid, pregnanolone hemisuccinate, produces sedation and neuroprotection in mice and rats (132). Weaver et al. (132) demonstrated that pregnanolone hemisuccinate (PAHS) inhibits NMDA-induced currents and cell death in primary cultures of hippocampal neurons. Additionally, administration of a non-sedating dose of PAHS to rats following focal cerebral ischemia reduces cortical and subcortical infarct size (Figure 13).

Ganaxolone (3-hydroxy-3-methyl-5-pregnane-20-one), an orally bioavailable synthetic analog of ALLO, is a positive allosteric modulator of GABA_A_R with promising basic and early-stage clinical outcomes as a potential novel treatment for PTSD (388, 390–392). Based on the mechanism of action of ganaxolone and work in preclinical animal models suggesting improved spatial memory function in an animal model of Angelman Syndrome (393), future investigation looking at the clinical value of this agent in anxiety disorders appears warranted. Another synthetic analog of ALLO, 3β-ethenyl-3α-hydroxy-5α-pregnan-20-one (Co 3-0593), has been found to have anxiolytic effects comparable to benzodiazepines after both subcutaneous and oral administration in rodents. An absence of tolerance to this synthetic neuroactive steroid is suggested by the observation that its effects were maintained with chronic administration (394).

Current research into the function of PregS is impeded by the lack of selective/specific antagonists and by a lack of knowledge of validated binding site(s). These drawbacks are further exacerbated by the susceptibility of the essential 3-hydroxysulfate to hydrolysis by sulfatases. To overcome these limitations, we and others are using PregS analogs to decipher mechanistic aspects of PregS-mediated effects on learning and memory function. We recently reported that low nanomolar concentration of PregS induce a delayed-onset increase of the neuronal response to NMDA and trafficking of NMDAR to the cell surface through an intracellular ([Ca^2+^]i)-dependent mechanism (47) (Figure 2). Moreover, we have demonstrated that low picomolar PregS increases [Ca^2+^]i and CREB phosphorylation and the frequency of spontaneous excitatory post-synaptic currents (48) (Figures 8, 9). More work is needed to determine if the synthetic analog of PregS, PREG hemisuccinate, has the potential to overcome the limitations associated with systemic administration of PregS.

## Conclusions

**Figure 14:**
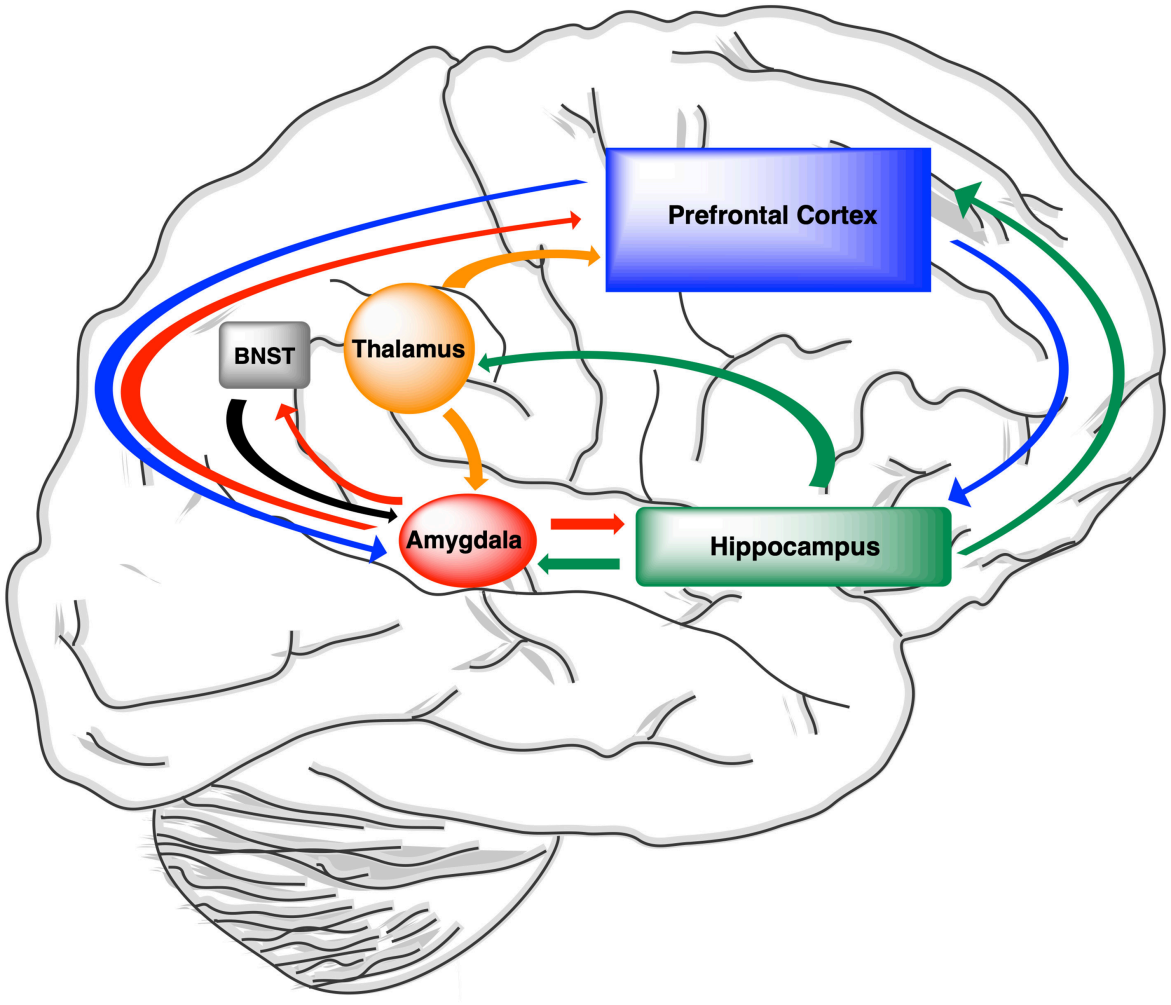
Pharmacologic connectivity pathways implicated in neurosteroidal modulation of memory function. Neural networks that project to and/or share reciprocal connections with the hippocampus are modulated by neurosteroids as well as neurotransmitters. As a result, learning and memory deficits are associated with many of neurologic and neuropsychiatric disorders in which neurosteroids are implicated. The amygdala (shown in red), which is implicated in anxiety disorders, shares reciprocal connections with the hippocampus (shown in green). Progesterone, which is metabolized to ALLO, modulates emotional memory function by influencing amygdalar activity (64). The effects of neurosteroids on learning and memory for fear-inducing stimuli appears to be different in males and females which show different brain levels of ALLO at baseline as well (395). Neuroactive steroids, such as ALLO that enhance inhibitory neurotransmission can provide symptomatic relief from anxiety by reducing intra-network connectivity in the salience network and the amygdala (75, 192, 290, 291). ALLO induces an increase in functional connectivity between the amygdala and prefrontal cortex (shown in blue), which is involved in processing of complex social and non-social stimuli. However, the increased inhibition associated higher levels of ALLO can also interfere with episodic memory function which depends on intact functional connectivity between the hippocampus and the pre-cuneus (not shown) (203). Interestingly, both PREG, which is metabolized to ALLO, and PregS, which is a positive modulator of NMDARs, improve working memory function in patients with schizophrenia in which cortical disinhibition due to hypofunction of excitatory NMDARs on PV interneurons in the prefrontal cortex has been implicated (51, 52, 65, 237, 238). Parahippocampal and hippocampal structures including, the trisynaptic circuit receives sensory and emotional inputs from sensory modalities via the thalamus (shown in orange) and the amygdala, respectively (396–398). The impact of powerful emotion-evoking stimuli are state independent when ALLO is injected into the amygdala and hippocampus, but state-dependent when it is injected into the BNST (shown in gray) (246). The response of the hippocampal trisynaptic circuit to neurosteroids also depends on disease state-dependent changes in neurosteroid biosynthesis and receptor expression (399–401), as well as age-related changes in steroid hormone levels which converge to influence the responsivity of this circuit to endogenous and exogenous sources of neurosteroids and their synthetic analogs (229, 401–403).

The effects of neurosteroids in memory function in neuropsychiatric and neurologic disorders reflect their modulatory interactions exerted via selective binding at the amino and transmembrane domains of specific subunits comprising GABA and glutamate receptors, among others. Age- and disease state-dependent changes in endogenous levels of neurosteroids appear to play a role in the emergence of the unique functional imbalances implicated in specific neuropsychiatric disorders and the associated memory deficits, which are mediated in part by changes in neural network activity within specific brain regions implicated in the encoding, consolidation, and retrieval of memories. It may be helpful to think of these findings in terms of a *pharmacological connectome* that reflects the interactions of neurosteroids with various neural networks involved with the encoding and recall of memories. We believe that a neural circuitry framework will help to guide future investigations into the potential role of neurosteroids and their synthetic analogs as neurotherapeutics for memory dysfunction (Figure 14).

## Author Contributions

All authors listed have made a substantial, direct and intellectual contribution to the work, and approved it for publication.

### Conflict of Interest Statement

The authors declare that the research was conducted in the absence of any commercial or financial relationships that could be construed as a potential conflict of interest.

## Abbreviations

ACTH: adrenocorticotropic hormone
AD: Alzheimer's disease
ALLO: allopregnanolone
AMPA: α-amino-3-hydroxy-5-methyl-4-isoxazolepropionic acid
ANT: adenine nucleotide transporter protein
BBB: blood-brain barrier
BNST: bed nucleus of the stria terminalis
CB1: cannabinoid receptor 1
CICR: Ca^2+^-induced Ca^2+^ release
CNS: central nervous system
DA: dopamine
DAT: dopamine transporter
DAT KO: dopamine transporter knockout
DHEA: dehydroepiandrosterone
DHEAS: dehydroepiandrosterone sulfate
GABA: gamma-aminobutyric acid
GAD: generalized anxiety disorder
GLIC: Gloeobacter ligand-gated ion channels
GnRH: gonadotrophin-releasing hormone
HPA axis: hypothalamic-pituitary-adrenal axis
HRSA: Hamilton Rating Scale for Anxiety
IL-6: interleukin-6
L-Type VGCC: L-type voltage-gated Ca^2+^ channel
LTD: long-term depression
LTP: long-term potentiation
MCI: mild cognitive impairment
NMDA: N-methyl-D-aspartate
OATPs: organic anion transporting peptides
ORAI1: Ca^2+^ release-activated Ca^2+^ channel protein 1
PAHS: pregnanolone hemisuccinate
pCREB: phosphorylated CREB
PET: positron emission tomography
PCOS: polycystic ovary syndrome
PREG: pregnenolone
PregS: pregnenolone sulfate
PTSD: post-traumatic stress disorder
PV: parvalbumin
PXR: pregnane xenobiotic receptor
P450 scc: cytochrome P450 side chain cleavage
sEPSC: spontaneous excitatory post-synaptic currents
5-HT: serotonin
SSRIs: selective serotonin reuptake inhibitors
StAR protein: steroidogenic acute regulatory protein
STIM1: stromal interaction molecule 1
THDOC: tetrahydrodeoxycorticosterone
TRH: thyrotropin-releasing hormone
TRP channels: transient receptor potential channels
TSPO: translocator protein
VDAC: voltage-dependent anion channel.
